# Pyruvate carboxylase promotes thyroid cancer aggressiveness through fatty acid synthesis

**DOI:** 10.1186/s12885-021-08499-9

**Published:** 2021-06-22

**Authors:** Chang Liu, Xiang Zhou, Yu Pan, Yang Liu, Yifan Zhang

**Affiliations:** 1 Department of Nuclear Medicine, Ruijin Hospital, Shanghai Jiao Tong University School of Medicine, No. 197, Ruijin 2nd Road, Shanghai, 200025 China; 2 Department of Nuclear Medicine, Renji Hospital, Shanghai Jiao Tong University School of Medicine, Shanghai, China

**Keywords:** Pyruvate carboxylase, Thyroid cancer, Fatty acid synthase, Lymph node metastasis, Proliferation

## Abstract

**Background:**

Pyruvate carboxylase (PC) is an important anaplerotic enzyme in the tricarboxylic acid cycle (TCA) in cancer cells. Although PC overexpression has been observed in thyroid cancer (TC), the mechanisms involved in the carcinogenic effects of PC are still unclear.

**Methods:**

Bioinformatics analysis and clinical specimens were used to analyze the relationship of PC expression with clinicopathological variables in TC. Fatty acid synthesis was monitored by LC/MS, Nile red staining, and triglyceride analysis. Mitochondrial oxygen consumption was evaluated by the Seahorse XF Mito Cell Stress Test. The correlation of PC with FASN and SREBP1c was assessed by qRT-PCR and IHC in 38 human TC tissues. Western blotting was used to evaluate the protein expression of PC, FASN, and SREBP1c and members of the AKT/mTOR and EMT pathways in TC cell lines. Wound-healing, CCK-8, and Transwell assays and a nude mouse xenograft model were used to verify the regulatory effects of PC and SREBP1c on thyroid tumor cell proliferation, migration and invasion.

**Results:**

We demonstrated that PC increased fatty acid synthesis, which then promoted TC progression and metastasis. Analysis of GEO data showed that the overexpression of PC in papillary thyroid cancer (PTC) was associated with PTC invasion and the fatty acid synthesis pathway. Analysis of clinical tissue specimens from PTC patients revealed that PC was more highly expressed in specimens from PTC patients with lymph node metastasis than in those from patients without metastasis. Multiple genes in the fatty acid synthesis signaling pathway, including FASN and SREBP1c, were downregulated in PC-knockdown TC cells compared to control cells. Lipid levels were also decreased in the PC-knockdown TC cells. Moreover, the ability of cells to grow, invade, and metastasize was also suppressed upon PC knockdown, suggesting that PC-mediated lipogenesis activation increases the aggressiveness of TC cells. In addition, PC was found to activate the AKT/mTOR pathway, thus improving FASN-mediated de novo lipogenesis in TC cells by upregulating SREBP1c expression. Studies in a nude mouse xenograft model showed that PC knockdown decreased tumor weight, but this effect was attenuated by forced expression of SREBP1c.

**Conclusions:**

Our results demonstrate that PC is strongly involved in the tumor aggressiveness of TC via its stimulation of fatty acid synthesis.

**Supplementary Information:**

The online version contains supplementary material available at 10.1186/s12885-021-08499-9.

## Introduction

Thyroid cancer (TC) has the highest prevalence among endocrine carcinomas worldwide [1–4]. However, 85% of TC cases are papillary thyroid cancer (PTC), which generally has a good prognosis, with a 10-year survival rate above 90% [5, 6]. However, aggressive variants of PTC characterized by invasion and metastasis can still threaten the quality of life of patients, and the 5-year disease-free survival rate for these variants is significantly lower than that for classical PTC [7–9]. In addition, anaplastic TC (ATC), which accounts for 2% of all TC patients, is one of the most aggressive malignancies and has a poor median survival time (3–5 months) [10, 11]. The clinical characteristics of early ATC include adjacent organ invasion and/or distant metastasis [12, 13]. Advanced PTC and ATC present challenges in the treatment of TC, and the mechanism of the malignant proliferation of advanced PTC and ATC is still not well understood.

Metabolic reprogramming can support tumor progression [14], which is involved in fatty acid metabolism and the TCA cycle [15]. The de novo synthesis of fatty acids provides components that improve the integrity of the cell membrane and provides signaling molecules for rapidly proliferating tumor cells, and this pathway does not rely on the uptake of exogenous fatty acids [16]. Some studies have reported that inhibiting cancer cell fatty acid availability can suppress cellular proliferation by targeting key lipogenesis-related enzymes such as fatty acid synthase (FASN) and acetyl-CoA carboxylase1 (ACC1) [17–23]. On the other hand, the TCA cycle provides biosynthetic precursor molecules that promote tumor progression [24]. To sustain the TCA cycle, tumor cells replenish TCA intermediates through anaplerotic pathways [25], such as the PC reaction [26–30], which converts carboxylated pyruvate into oxaloacetate, which is used as a substrate for biosynthesis [31–33]. PC-replenished TCA promotes the growth of human TC cells [30]. However, whether there is a correlation between the carcinogenic effects of PC and fatty acid synthesis is still elusive.

## Materials and methods

### Gene expression data acquisition and bioinformatic analysis

An expression profiling array dataset (GSE6004) from the Gene Expression Omnibus (GEO) databases was analyzed by the Affymetrix Human Genome Array with the annotation platform GPL570 [HG-U133_plus_2]. The GSE6004 dataset contains 7 samples from 7 patients with invasive PTC and 4 control thyroid tissues. The detailed information is shown in Table 1. Using R packages, we assessed GSE6004 RAW datasets by background correction, normalization, expression calculation, and probe integration. The robust multi-array average (RMA) and mismatch probe (PM) approaches were used for processing the datasets. The *P*-values were adjusted by the Benjamini-Hochberg method, and the fold-changes (FC) were calculated by the false discovery rate (FDR) procedure. Differentially expressed genes (DEGs) in PTC with |log2-fold change| > 1 and *p* < 0.05 were selected. Gene Ontology (GO) and Kyoto Encyclopedia of Genes and Genomes (KEGG, http://www.kegg.jp/ or http://www.genome.jp/kegg) pathway enrichment analyses of the DEGs were carried out using the online Database for Annotation, Visualization, and Integrated Discovery (DAVID) Gene Functional Classification Tool (http://david.abcc.ncifcrf.gov/).

**Table 1:** Basic features of the GSE6004 database

GEO no.	Subtype
GSM 139002	Normal thyroid
GSM 139003	Normal thyroid
GSM 139004	Normal thyroid
GSM 139005	Normal thyroid
GSM 139013	Thyroid cancer invasive area
GSM 139014	Thyroid cancer invasive area
GSM 139015	Thyroid cancer invasive area
GSM 139016	Thyroid cancer invasive area
GSM 139017	Thyroid cancer invasive area
GSM 139018	Thyroid cancer invasive area
GSM 139019	Thyroid cancer invasive area

*GSE* gene set enrichment, *GEO* gene expression omnibus

### Patients

Thirty-eight surgical specimens from PTC patients after total thyroidectomy and central nodal dissection with final tissue sample confirmation were collected from July 2018 to November 2018, and all patients signed informed consent forms. The study design was approved by the Ethical Review Board of Ruijin Hospital, Shanghai Jiao Tong University School of Medicine. Clinical characteristics, including age, sex, tumor size, clinical stage, and lymph node status, are shown in Table 3.

### Cell culture and reagents

The human TC cell lines TPC1 and 8505C, derived from PTC and ATC, respectively, were cultured with RPMI-1640 (Gibco, Thermo Scientific, MA, USA) medium containing 10% fetal bovine serum (FBS) (Gibco, Thermo Scientific, MA, USA) and 1% penicillin at 37 °C with 5% CO_2_. LY294002 (Biogems International, CA, USA), Lipofectamine 2000 (Invitrogen, CA, USA), Nile red (Santa Cruz Biotechnology, Texas, USA), G418 (GenePharma, Shanghai, China), puromycin (Gibco, Thermo Scientific, MA, USA), and polybrene (GenePharma, Shanghai, China) were also used in this study. The antibodies used were anti-PC (Santa Cruz Biotechnology, TX, USA), anti-SREBP1c (Affinity Biosciences, OH, USA), anti-FASN (Abcepta, CA, USA), anti-phospho-mTOR (Santa Cruz Biotechnology, TX, USA), anti-mTOR (Proteintech Group, IL, USA), and anti-phospho-Akt (Cell Signaling Technology, MA, USA). Antibodies against ACC1 (Proteintech Group, IL, USA), ACLY (Epigentek, NY, USA), E-cadherin (Affinity Biosciences, OH, USA), Vimentin (Affinity Biosciences, OH, USA), snail1 (Proteintech Group, IL, USA), ZEB2 (Proteintech Group, IL, USA), GAPDH (Beyotime Biotechnology, Shanghai, China), and α-Tubulin (Beyotime Biotechnology, Shanghai, China) were also used in this study.

### Cell transfections

Cells were seeded in 6-well plates (1 × 10^5^ cells/well). Lipofectamine 2000 (Invitrogen, CA, USA) was used for transfection of siRNA-SREBP1c and the control siRNA, which were purchased from Pharma China. Lentiviral vectors containing PC short hairpin RNA (shRNA), the coding sequence of PC (NM_000920.3), SREBP1c cDNA, the coding sequence of SREBP1c (NM_001321096.3), and their controls were purchased from Hanbio (Shanghai, China) and transfected into TC cells. Stable lentivirus-infected TC cells were cultured in complete medium and screened with 1 μg/ml puromycin. The sequences of the forced expression and knockdown constructs used in TC cells are shown in Table 2.

**Table 2:** Sequence of primers and siRNA used in the study

Gene	Prime (5′ → 3′)
**Primers used in RT-PCR analysis**	
PC	Forward: ATGTTGCCCACAACTTCAGCAAGC
	Reverse: AGTTGAGGGAGTCAAACACACGGA
SREBP1c	Forward: GGAGCCATGGATTGCACATT
	Reverse: CAGGAAGGCTTCCAGAGAGG
**FASN**	Forward: GGTCTTGAGAGATGGCTTGC
	Reverse: AATTGGCAAAGCCGTAGTTG
ACC	Forward: GATCAAACTCTGGGAGTCTATG
	Reverse: TCTCGGCCTTCTGGATATT
ACLY	Forward: CAACTTTGCCTCTCTCCGCTCTG
	Reverse: TCCCTTCTGGTCCGCCTTCTTG
β-actin	Forward: GCACCACACCTTCTACAATG
	Reverse: TGCTTGCTGATCCACATCTG
**Primers used in siRNAs**	
siCtrl	Forward: UUCUCCGAACGUGUCACGUTT
	Reverse: ACGUGACACGUUCGGAGAATT
siSREBP1c	Forward: GCAAGGCCAUCGACUACAUTT
	Reverse: AUGUAGUCGAUGGCCUUGCTT
**Primers used in gene cloning**	
SREBP1c	Forward: TCCGGTGAATTCCTCGAGCCACCATGGATTGCACTTTCGAAGAC
	Reverse: TCGCAGATCCTTGGATCCCTAGCTGGAAGTGACAGTGGTC
PC	Forward: GATCCGCCAGACAACGTGGTCTTCAAGTTCTTTCAAGAGAA GAACTTGAAGACCACGTTGTCTGGTTTTTTC
	Reverse: AATTGAAAAAACCAGACAACGTGGTCTTCAAGTTCTTCTCTT GAAAGAACTTGAAGACCACGTTGTCTGGCG

*PC* Pyruvate carboxylase, *SREBP1c* sterol regulatory element-binding protein 1c, *FASN* fatty acid synthesis, *ACC* acetyl-CoA carboxylase, *ACLY* ATP citrate lyase, *siCtrl* control small interfering RNA

### Analysis of fatty acids by liquid chromatography/mass spectrometry (LC/MS)

To extract intracellular metabolites, 1 × 10^7^ cells were collected and assessed by LC-MS/MS. LC was performed using a Waters ACQUITY UPLC system with an ACQUITY BEH Amide column. Eluent A was 0.1% ammonia, whereas eluent B was 100% acetonitrile and 0.1% ammonia (gradient elution) at a flow rate of 0.5 ml/min. MS was performed on a 6500 QTRAP mass spectrometer (AB ACIEX, Foster City, CA, USA) and carried out at an ion spray voltage of − 4.500 kV and a turbo spray temperature of 300 °C with the electrospray ionization (ESI) ion source in negative mode. The contents of fatty acids in TC cells were quantitatively determined with the multiple reaction monitoring (MRM) mode. The ion pairs for oleic acid, stearic acid, palmitoleic acid, and palmitic acid were 281.2/126.8, 253.4/126.8, 283.5/265.4, and 255.4/237.4; the declustering potentials (DPs) were − 105 V, − 100 V, − 105 V, and − 105 V′ and the collision energies (CEs) were − 35 eV, − 35 eV, − 15 eV, and − 30 eV, respectively.

### Nile red staining

The lipophilic fluorescence dye Nile red (Santa Cruz Biotechnology, Inc., USA) was used to monitor the cytoplasmic membranes and lipid droplets (LDs) in TC cells by fluorescence microscopy and flow cytometry. Cells were fixed with 4% paraformaldehyde for 30 min at room temperature and then washed with 1 × PBS three times. Then, the cells were incubated with 1 μg/ml Nile red for 15 min at 37 °C and washed with 1 × PBS three times. DAPI staining solution (Beyotime Biotechnology, Shanghai, China) was used to counterstain the nuclei. The images were recorded by an inverted fluorescence microscope with a 40× objective lens (Carl Zeiss, Germany). Alternatively, cells were harvested and suspended at 1 × 10^5^ cells/ml. The suspended cells were stained with 1 μg/ml Nile red for 10 min at 37 °C and immediately analyzed with a phycoerythrin (PE) signal detector using a CytoFLEX cytometer (Beckman-Coulter, USA). Data are reported as the product of the fluorescence intensity (FI) multiplied by the percentage of gated cells to avoid discrepancies between time points due to the setup and the compensation performed for every time point. Unstained cells were used to adjust the instrument settings to account for cellular autofluorescence or background fluorescence.

Increased Nile red fluorescence, relative to that of the control samples, indicated intracellular phospholipid staining of cells. In order to compare the changes in intracellular Nile red staining, statistical analysis was performed on the mean fluorescence intensity data to determine variability and significant differences between experimental treatment and vehicle control treatment.

### Triglyceride (TAG) analysis

Chloroform/methanol (2:1) extraction was used to extract triglycerides from cell homogenates, and the intracellular triglyceride content was then quantitatively evaluated with an EnzyChrom™ Triglyceride Assay Kit (Bioassay Systems, Hayward, CA) following the instructions of the kit [34–36].

### Fatty acid oxidation analysis

The Cell Mito Stress Test can detect changes in the mitochondrial oxygen consumption rate (OCR) under oligomycin treatment, reflecting fatty acid oxidation ability in real time by comparing the level of exogenous palmitic acid with that of its solvent BSA [37, 38]. 8505C cells were cultured overnight in an XF24 Cell Culture Microplate (Agilent Technologies, UK) at a density of 1 × 10^5^ and then detected by a Seahorse XF24 Extracellular Flux Analyzer (Agilent Technologies, UK).

### Quantitative reverse transcription PCR (qRT-PCR)

Total RNA was separated using TRIzol (Sangon Biotech, Shanghai, China), reverse transcribed into cDNA following the protocol of PrimeScript™ RT Master Mix (TaKaRa, Japan), incubated at 37 °C for 15 min, 85 °C for 5 s, and then cooled to 4 °C. qRT-PCR was performed following the protocol for SYBR Green PCR Master Mix (TaKaRa) on the StepOnePlus Real-Time PCR System (Applied Biosystems, USA). The temperature cycling protocol consisted of 30 s denaturation at 95 °C, followed by 40 cycles of 95 °C for 5 s and 60 °C for 34 s. The 40 cycles were followed by melting curve analysis: 15 s at 95 °C, 60 °C for 1 min, and 95 °C for 15 s. The reference gene was human β-actin. The 2^-ΔΔCt^ method was used to evaluate the expression levels of target genes. The primer sequences used in this study are shown in Table 2.

### Western blotting

Cells were lysed with RIPA buffer (Beyotime, Shanghai, China) on ice for 30 min. The protein concentration was measured using a BCA protein assay kit (Thermo Scientific Pierce, Sunnyvale, CA). Protein was loaded on a 6% SDS-PAGE gel and transferred onto polyvinylidene fluoride (PVDF) membranes (Millipore, MA, USA). After blocking, the cells were incubated at 4 °C overnight with primary antibodies and then incubated with secondary antibody 1 h after three washes with 1 × TBST (Tris-buffered saline and Tween 20). Finally, the membranes were washed three times with TBST and detected by the Enhanced Chemiluminescence Kit (Life Technologies). The reference antibody was human anti-GAPDH.

### Immunohistochemistry (IHC)

Thirty-eight PTC tissue samples from patients who had histologically confirmed PTC were obtained from Ruijin Hospital affiliated with Shanghai Jiao Tong University School of Medicine, Shanghai, China. All patients signed informed consent forms, and this research was approved by the ethics committee of the Shanghai Jiao Tong University School of Medicine. The clinical characteristics of the patients are listed in Table 3. The PTC tissues were fixed in 4% paraformaldehyde and then embedded in paraffin. The intensity score of target proteins was independently evaluated by two pathologists without prior knowledge about the patient and sample (200-fold magnification). The percentage of positively stained tumor cells was scored as follows: < 10%, 0; 10–25%, 1; 26–50%, 2; 51–75%, 3; and 75%, 4. The intensity of the positive tumor cells was scored as follows: 0 (negative), 1 (weak), 2 (moderate), 3 (moderately strong), or 4 (strong). The percentage and intensity scores were multiplied to obtain a final score for target proteins in PTC tissues, which ranged from 0 to 16. A final score < 2 indicated negative (−) expression, a score ≥ 2 to < 7 indicated weakly positive (+) expression, a score ≥ 7 to < 12 indicated moderately positive (++) expression, and a score ≥ 12 indicated strongly positive (+++) expression.

**Table 3:** Correlations between PC mRNA expression in PTC tissues and Clinicopathological features of 38 patients with PTC

Clinicopathological		PC mRNA		
	n	Median ^a^ (IR)	*Ζ*	*P-*value
Age (years)				
< 55	31	0.192 [0.059 ~ 0.351]	−1.412	0.167
≥ 55	7	0.389 [0.149 ~ 2.321]		
Sexual				
Male	12	0.354 [0.140 ~ 1.944]	−2.324	0.020*
Female	26	0.149 [0.029 ~ 0.313]		
TNM				
I-II	14	0.137 [0.044 ~ 0.350]	−1.422	0.155
III-IV	24	0.304 [0.096 ~ 0.980]		
Lymph node metastasis				
with	24	0.310 [0.134 ~ 1.069]	−2.330	0.019*
without	14	0.102 [0.029 ~ 0.193]		

*P* values were calculated using Mann-Whitney test.

*n* number of patients for each studied variable, *IR* interquartile range

*, statistically significant

^a^ median of PC mRNA expression level

### Cell counting kit (CCK-8) assay

Cell survival was measured using the CCK-8 assay (Beybond, China). Cells were seeded onto 96-well plates at a density of 1 × 10^3^ cells/well. After 24 h, 10 μl cholecystokinin-8 was added to each well and incubated for 2 h. Then, a microplate reader was used to read the absorbance at 450 nm.

### Cell migration and invasion assay

Cell migration was observed using wound healing and Transwell assays. The steps of the wound healing assay were as follows: cells were seeded in a 6-well plate at a density of 1 × 10^6^ cells/ml and cultured until they filled the culture dish. A 200-μl pipette tip was used to draw a vertical line on the culture dish, and the cells were cultured for another 48 h. Photographs were taken with a microscope (Carl Zeiss, Germany), and changes in the widths of the scratches were recorded. Transwell assays were carried out as follows: a Transwell chamber (Costar, USA) was placed into a 24-well plate, 200 μl 1640 medium containing 5 × 10^5^ cells without FBS was added, and 500 μl 1640 medium with 10% FBS was added to the 24-well plate. After further culturing for 24 h, the 1640 media was discarded, the polycarbonate membrane was fixed in 4% paraformaldehyde, and then the polycarbonate membrane was stained with 1% crystal violet. Photographs were taken with a microscope (Carl Zeiss, Germany), and the number of cells in the lower chamber was recorded.

Cell invasion was observed using Transwell assays. Transwell assays were carried out as follows: a Transwell chamber equipped with Matrigel (Costar, USA) was placed into a 24-well plate, 500 μl 1640 medium containing 2.5 × 10^5^ cells without FBS was added, and 500 μl 1640 medium with 20% FBS was added to the 24-well plate. After further culturing for 48 h, the 1640 media was discarded, the polycarbonate membrane was fixed in 4% paraformaldehyde, and then the polycarbonate membrane was stained with 1% crystal violet. Photographs were taken with a microscope (Carl Zeiss, Germany), and the number of cells in the lower chamber was recorded.

### Animal experiments

8505c cells infected with or without different lentiviral vectors was conducted as described above. A total of 15 female 4-week-old BALB/c nude mice (12–14 g) from SPF Laboratory of Charles River (Shanghai, China) and randomly divided into the following three groups with 5 mice each: PC-WT, PC-sh, and PC-sh + SREBP1c OE. A total of 1 × 10^7^ cells in 100 μl PBS were subcutaneously injected into the right armpits of the mice. Tumor volume was monitored with digital calipers for 10 days and calculated using the formula: (length × width × width)/2. A time-volume curve was generated to study the growth of xenografts. Before the diameter of the tumor exceeded 15 mm, mice were anesthetized with 2% isoflurane (Ningfen, Shandong, China) and sacrificed by cervical dislocation, and the primary tumor was excised, weighed, and embedded in paraffin. All animal experimental procedures were approved by the Animal Ethics Committee of Ruijin Hospital.

### Statistical analysis

Results data were presented as the mean ± SD. The Mann-Whitney *U* test was used to evaluate unpaired differences between two groups. Spearman rank correlation analysis was performed to evaluate the relationship between PC expression levels and fatty acid synthesis-related enzyme expression levels. Differences for which *p* < 0.05 indicated statistical significance. All statistical analyses were assessed using SPSS 22.0.

## Results

### *PC* is highly expressed in aggressive PTC tissues

In the GSE6004 dataset, PC was more highly expressed in invasive PTCs than in normal thyroid tissues, with a log_2_ FC of 1.85. Similarly, PC was more highly expressed in PTC center area tissues than in normal thyroid tissues, with a log_2_ FC of 1.83. However, PC levels were not significantly different between the central regions and invasive regions of PTC. Then, we further explored *PC* mRNA expression in surgical PTC tumor tissues. The results also showed that PC was more highly expressed in PTC with lymph node metastasis than in PTC without metastasis (0.310 [0.134 ~ 1.069] vs. 0.102 [0.029 ~ 0.193], *p* = 0.019). The results showed that PC is associated with TC aggressiveness.

### *PC* functions are associated with fatty acid synthesis

**Fig. 1:**
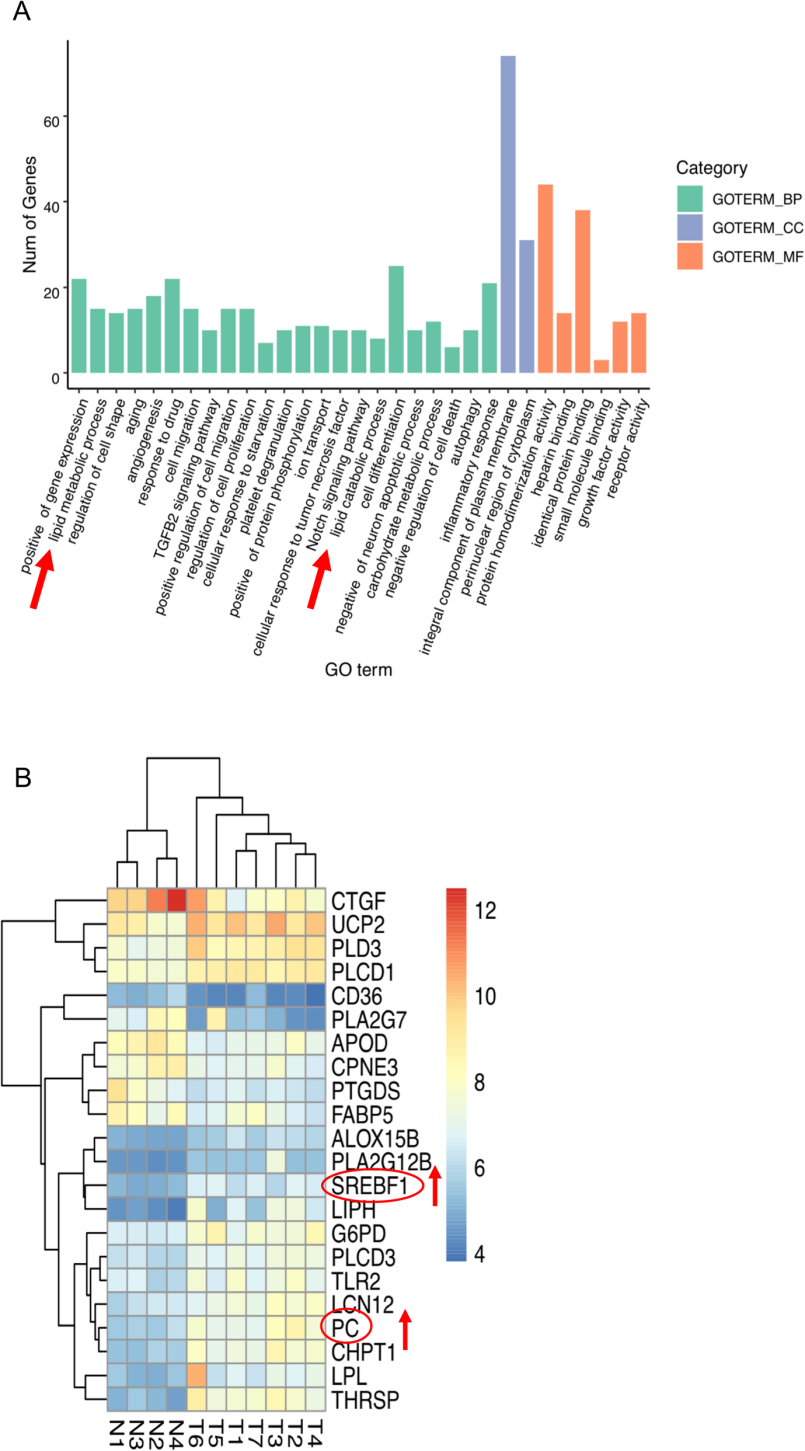
Basic features of the GSE6004 dataset. **A** GO analysis of the differential gene function enrichment between tumor tissue and normal control tissue. The red arrow represents fatty acid-related functions. **B** DEGs in fatty acid-related pathways; red indicates high expression in tumor tissues, and blue indicates low expression in tumor tissues. Note: GO (Gene Ontology), DEG (differentially expressed gene)

GO enrichment analysis showed that the DEGs between invasive PTC and normal tissues were clustered in various pathways, including cell differentiation, cell migration, the inflammatory response and the lipid metabolic process (shown in Fig. 1A). Additionally, both PC and SREBP1 were involved in the lipid metabolic process (shown in Fig. 1B).

### *PC* increases fatty acid synthesis in TC cells

**Fig. 2:**
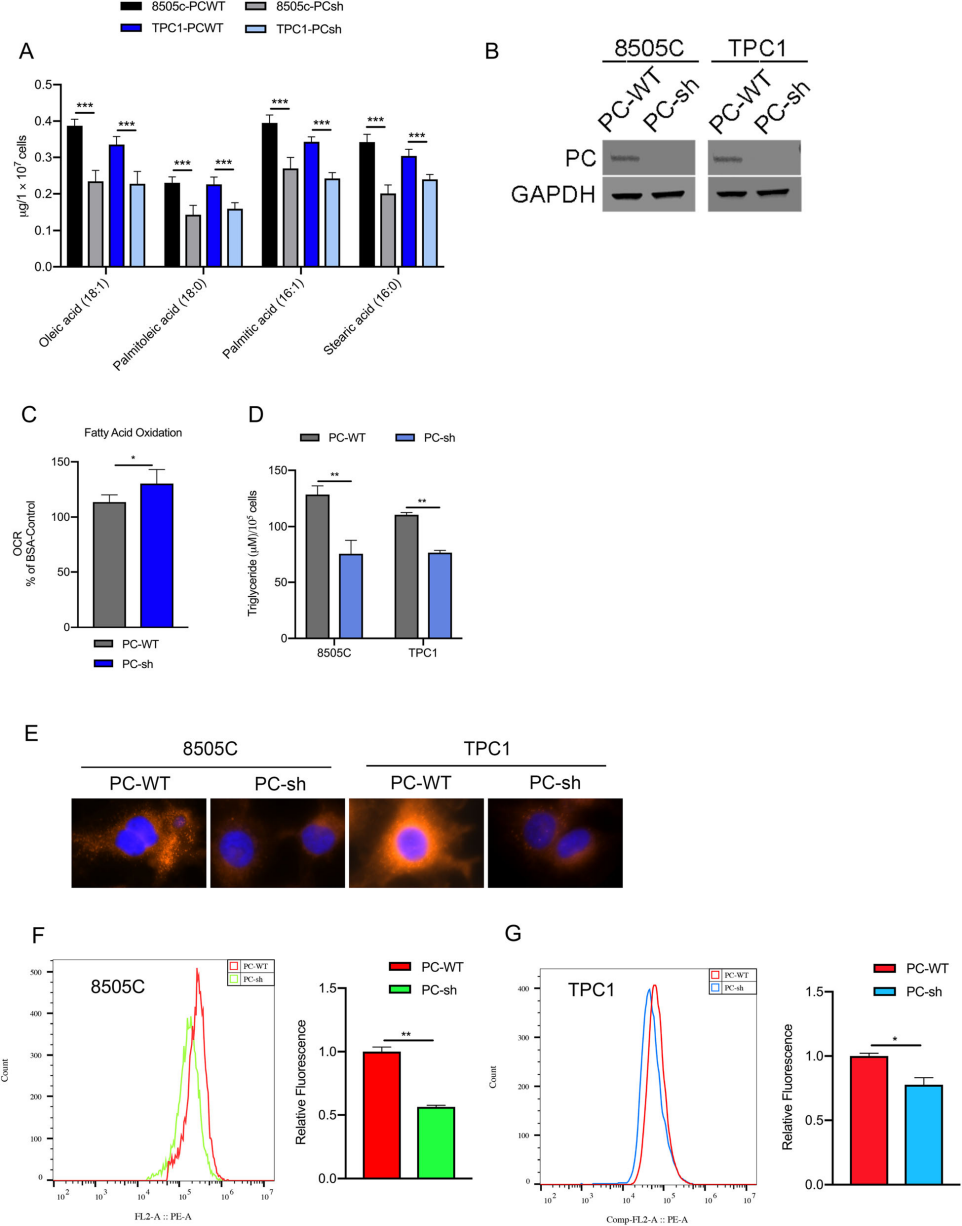
Effect of PC expression on lipid accumulation in TC cells. **A** The intracellular free acid content was quantified by LC/MS. **B** Western blotting was used to evaluate PC expression in TC cell lines as indicated. **C** Fatty acid oxidation analysis was detected in 8505C cells by a Seahorse XF24 Extracellular Flux Analyzer. **D** The cellular content of TAGs was measured in different TC cells. **E** Nile red dye and DAPI staining were used to detect the neutral lipid content in TC cells by microscopy with a 40× objective lens. **F**, **G** 8505C and TPC1 cells were stained with Nile red and subjected to flow cytometry analysis. The results are expressed as means ± standard deviation (SD) of three independent experiments and shown in histograms on the right. ^*^ *p* < 0.05; ^***^ *p* < 0.01 versus PC WT. Note: LC/MS (liquid chromatography/mass spectrometry), PC (pyruvate carboxylase), TC (thyroid cancer), TAG (tricarboxylic acid cycle)

We compared the lipid and free fatty acid contents between TC cell lines (8505C and TPC1) with PC knockdown (PC-sh) and those with normal expression of PC (PC-WT) to evaluate the role of PC in lipogenesis in TC cells (shown in Fig. 2B). The results showed that the contents of intracellular triglycerides (TAGs) were significantly decreased in PC-knockdown TC cells compared to control cells (shown in Fig. 2D). Staining with Nile red, a highly sensitive dye that can detect LDs in the cytoplasm, also showed that PC knockdown can decrease intracellular LD content in 8505C cells and TPC1 cells (shown in Fig. 2E-G). Metabolite analysis by LC/MS showed that PC-knockdown TC cells had significantly lower levels of free fatty acids, such as oleic acid (C18:1), stearic acid (C18:0), palmitoleate acid (C16:1) and palmitic acid (C16:0), than control cells (shown in Fig. 2A). To maintain lipid content and meet their high energy demands, tumor cells inhibit fatty acid oxidation (FAO) [39]. Thus, the rate of FAO was determined by an extracellular flux analyzer to assess the OCR, which mainly reflects mitochondrial oxidative phosphorylation activity. FAO analysis showed that inhibiting PC increased the oxidation of exogenously supplied palmitate (shown in Fig. 2C).

### *PC* activates the de novo synthesis of fatty acids by upregulating key lipogenic enzymes

**Fig. 3:**
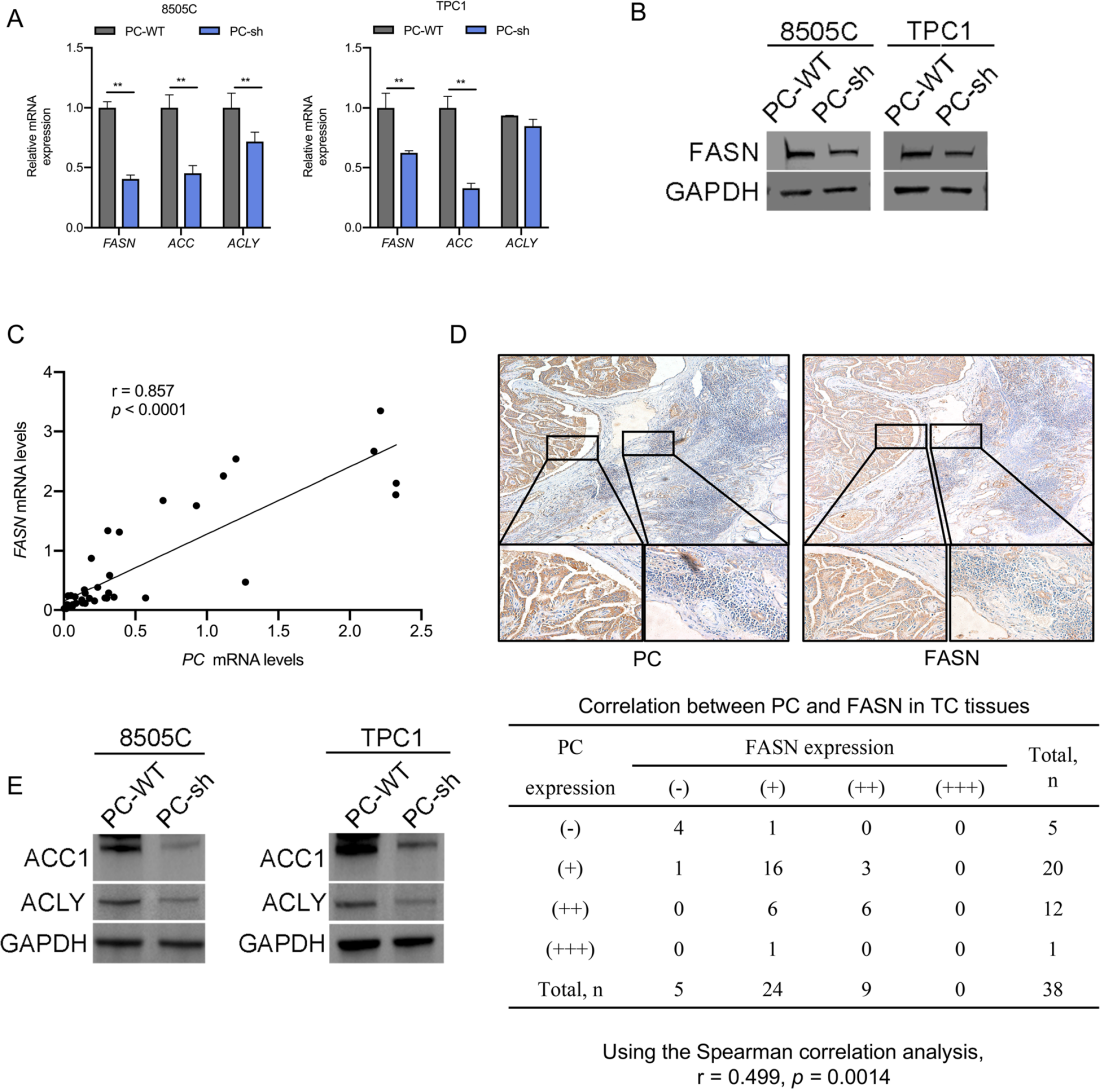
Upregulation of the expression of lipogenic enzymes by PC in TC cells. **A** qRT-PCR analysis of mRNA expression and the lipogenic enzyme genes *FASN*, *ACC1*, and *ACLY* in 8505C and TPC1 cells. **B** Western blot analysis of the protein level of the lipogenic enzyme *FASN* in the indicated cells, the full blot is provided in supplementary file. **C** Results from the correlation analysis between mRNA expression levels of *PC* and *FASN* in 38 TC tissues, as show in the scatter plot. **D** IHC staining was used to analyze the relationship between the protein expression levels of PC and FASN. **E** Western blot analysis of the protein level of the lipogenic enzymes *ACC1* and *ACLY* in the indicated cells; the full blot is provided in the supplementary file. Note: FASN (fatty acid synthesis), ACC1 (acetyl-CoA carboxylase), ACLY (ATP-citrate lyase), IHC (immunohistochemistry), qRT-PCR (quantitative reverse transcription PCR)

Based on the GSE6004 dataset, PC associated with SREBP1 was involved in the lipid metabolic process. One isoform of SREBP1, SREBP1c, plays an important role in the fatty acid synthesis pathway and enhances the transcription of lipogenic genes, such as *FASN, ACLY,* and *ACC1* [40, 41]. We analyzed the mRNA and protein expression of these enzymes in 8505C and TPC1 cells. The results showed that the mRNA expression levels of FASN, ACC1, and ACLY were significantly decreased in PC-knockdown 8505C cells compared to control cells with normal PC expression, but the mRNA expression of ACLY was not significantly decreased in TPC1 cells (Fig. 3A), and the protein level of FASN was also decreased in TC cells (Fig. 3B). In addition, we further investigated the relationship between the mRNA and protein expression of *PC* and *FASN* in 38 PTC tissue samples by RT-PCR and IHC. The results showed that there was a positive correlation between *PC* and *FASN* mRNA levels (*r* = 0.857, *p* < 0.0001) (shown in Fig. 3C). The IHC scores were assessed by Spearman’s rank correlation coefficients, and the results showed that there was a significant positive correlation between PC and FASN (*r* = 0.499, *p* = 0.0014) (shown in Fig. 3D). A positive correlation was also observed between *PC* and *ACC1* mRNA levels (*r* = 0.485, *p* = 0.0023) and *PC* and *ACLY* mRNA levels (*r* = 0.359, *p* = 0.027). The ACC1 and ACLY protein levels were also decreased in PC-knockdown 8505C cells compared to control cells with normal PC expression, but the ACLY protein showed no significant decrease in TPC1 cells (Fig. 3E). Then, we further analyzed the correlations between clinicopathological features and *ACC1*, *ACLY* and *FASN* mRNA levels in surgical PTC tumor tissues. However, the results showed that only *FASN* was more highly expressed in male PTC patients than in female patients (0.952 [0.240 ~ 1.919] vs. 0.203 [0.091 ~ 0.356], *p* = 0.016).

### *PC* promotes the expression of fatty acid synthesis enzymes by upregulating *SREBP1c*

**Fig. 4:**
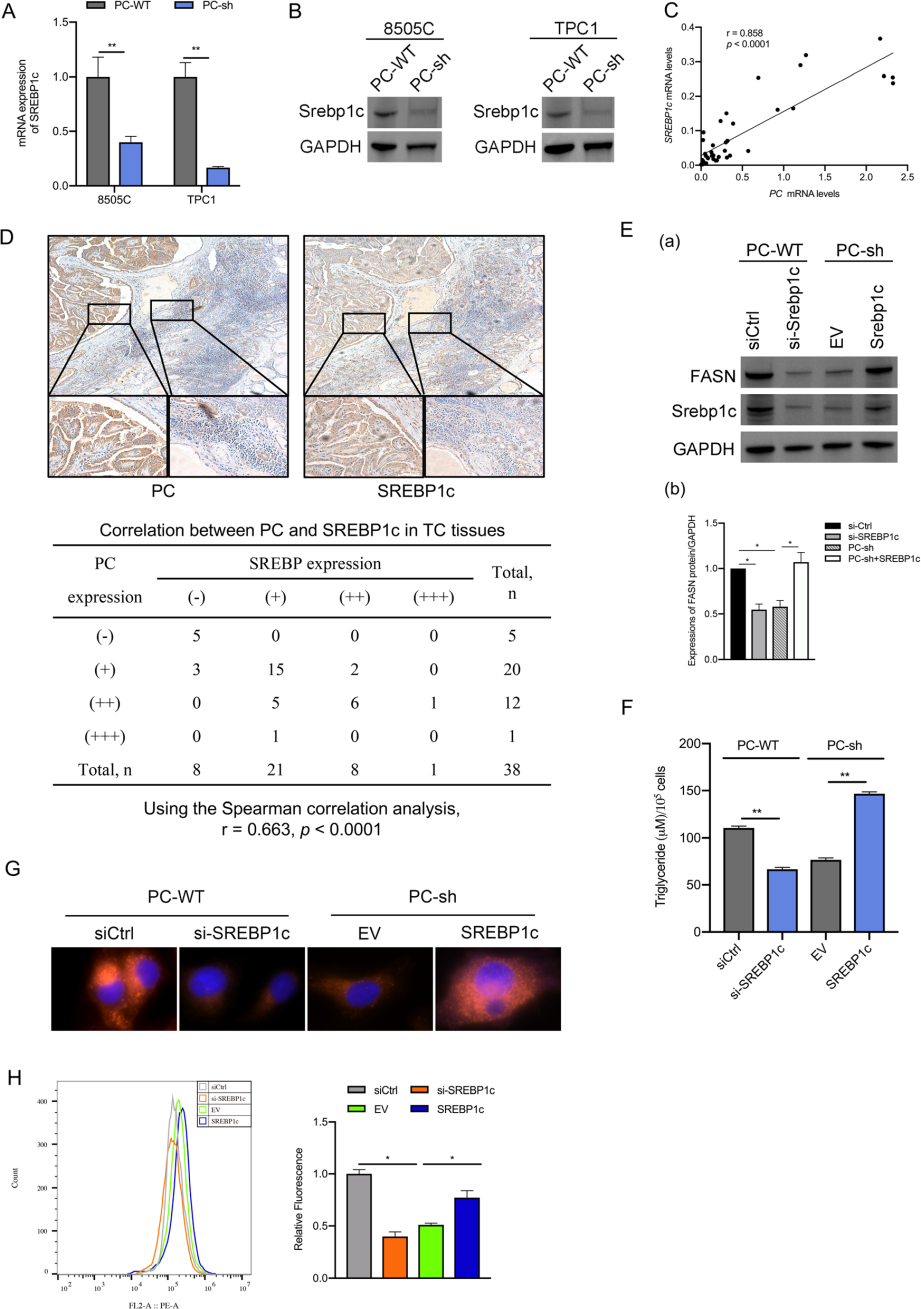
PC upregulates the expression of lipogenic enzymes by activating SREBP1c. **A**, **B** qRT-PCR and Western blot analysis of the mRNA and protein expression of *SREBP1c* in 8505C and TPC1 cells. **C** Results from the correlation analysis between the mRNA expression levels of *PC* and *SREBP1c* in 38 TC tissues, as shown in the scatter plot. **D** IHC staining was used to analyze the relationship between the protein expression of PC and SREBP1c. (**E**) a. Western blot analyses of SREBP1c and FASN in 8505C/PC-WT cells transfected with si-SREBP1c or control siRNA (siCtrl) and in 8505C/PC-sh cells overexpressing SREBP1c vector or empty vector (EV); the full blot is provided in supplementary file. b. Quantitative analysis of FASN protein expression. ^*^ *p* < 0.05, the si-Ctrl group compared with the si-SREBP1c and PC-sh group, the PC-sh group compared with the PC-sh + SREBp1c group. **F** The cellular TAG content was measured in 8505C cells. **G** Nile red dye and DAPI staining were used to detect the neutral lipid content in 8505C cells by microscopy with a 40× objective lens. **H** 8505C/PC-WT cells transfected with si-SREBP1c and 8505C/PC-sh cells overexpressing SREBP1c vector were stained with Nile red dye and subjected to flow cytometry analysis. The results are expressed as means ± standard deviation (SD) of three independent experiments and shown in histograms on the right. ^*^ *p* < 0.05; ^***^ *p* < 0.01 versus 8505C/PC-WT cells transfected with control siRNA (siCtrl) and 8505C/PC-sh cells overexpressing empty vector (EV). Note: SREBP (sterol regulatory element-binding protein)

Sterol regulatory elementary binding proteins (SREBPs) are members of the SREBP family of transcription factors, which include SREBP1a, SREBP1c, and SREBP2 [42–44]. It is well known that SREBPs have central roles in the biosynthesis of fatty acids by upregulating the expression of lipogenic enzymes [44]. In our study, bioinformatics analysis suggested that SREBP1 may play an important role in regulating PC-related lipogenesis. Several studies have demonstrated that overexpression of FASN in human breast cancer is induced by SREBP1c but not SREBP1a [45]. To confirm whether the role of PC-mediated upregulation of fatty acid synthesis enzymes occurred through SREBP1c, we first used RT-PCR and Western blotting to assess the mRNA and protein expression levels of SREBP1c in TC cell lines. The results showed that SREBP1c expression was decreased in PC-sh 8505C cells compared to PC-WT 8505C cells and TPC1 cells (shown in Fig. 4A, B). Second, we evaluated the mRNA expression levels of SREBP1c in 38 PTC tissue samples. Correlation analysis showed that there was a positive correlation between *PC* and *SREBP1c* mRNA levels (*r* = 0.858, *p* < 0.0001) (shown in Fig. 4C). IHC scores were assessed by Spearman’s rank correlation coefficients, and the results showed that there was a significant positive correlation between PC and SREBP1c (*r* = 0.663, *p* < 0.0001) (shown in Fig. 4D). Third, we analyzed whether PC upregulates the expression of FASN through SREBP1c. The results showed that the expression of FASN decreased in 8505C/PC-WT cells transfected with si-SREBP1c and increased in 8505C/PC-sh cells overexpressing SREBP1c (shown in Fig. 4E). Moreover, the intracellular TAG content and Nile red staining rate in 8505C cells were significantly decreased after SREBP1c knockdown, and SREBP1c overexpression in PC-knockdown cells increased intracellular TAG and LD levels (shown in Fig. 4F-H).

### *PC* induces the expression of *SREBP1c* via the *Akt/mTOR* signaling pathway in 8505C cells

**Fig. 5:**
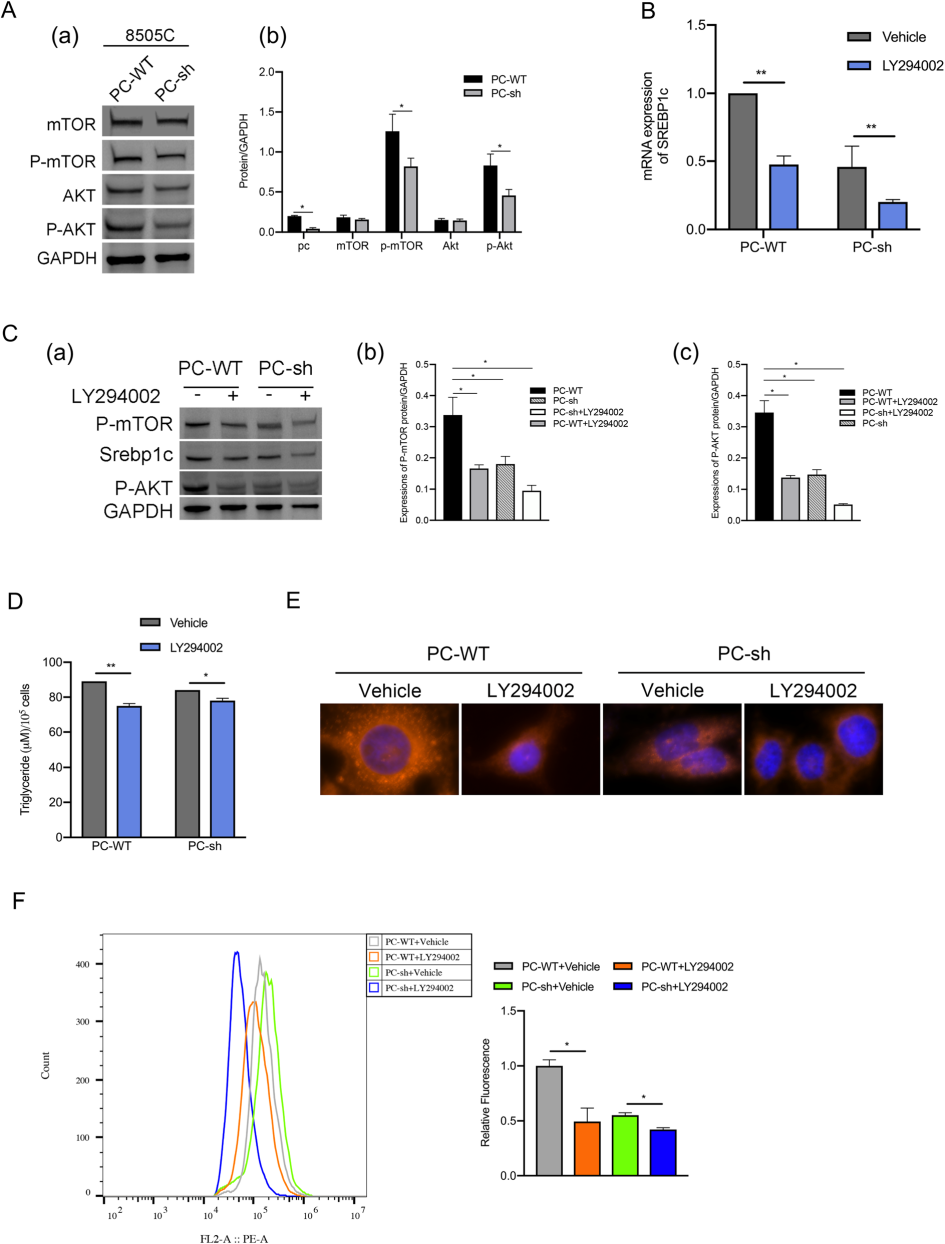
PC upregulates the expression of SREBP1c by activating the Akt/mTOR signaling pathway. (**A**) a. Western blot analysis of phosphorylated Akt (p-Akt), mTOR and phosphorylated mTOR (p-mTOR) in 8505C/PC-WT and 8505C/PC-sh cells; the full blot is provided in supplementary file. b. Quantitative analysis of the expression of each protein. ^*^ *p* < 0.05, compared with the PC-WT group. **B** qRT-PCR analyses of SREBP1c mRNA in different 8505C cells treated with the Akt inhibitor LY294002. (**C**) a. Western blot analysis of the protein expression of SREBP1c, p-Akt, and p-mTOR in different 8505C cells treated with an Akt inhibitor, LY294002; the full blot is provided in supplementary file. b. Quantitative analysis of p-mTOR protein expression. ^*^ *p* < 0.05, compared with the PC-WT group. c. Quantitative analysis of p-AKT protein expression. ^*^ *p* < 0.05, compared with the PC-WT group. **D** The cellular TAG content was measured in different 8505C cells. **E** Nile red dye and DAPI staining were used to detect the neutral lipid content in 8505C cells by microscopy with a 40× objective lens. **F** 8505C/PC-WT cells treated with LY294002 and 8505C/PC-sh cells treated with LY294002 were stained with Nile red dye and subjected to flow cytometry analysis. The results are expressed as means ± standard deviation (SD) of three independent experiments and shown in histograms on the right. ^*^ *p* < 0.05; ^***^ *p* < 0.01 versus 8505C/PC-WT cells treated with vehicle and 8505C/PC-sh cells treated with vehicle. Note: mTOR (mechanistic target of rapamycin)

According to reports in the literature, cancer cells need more fatty acids for cell membrane formation and rapid proliferation compared to normal cells [46], and they meet these needs by reprogramming metabolism through phosphatidylinositol 3-kinase (PI3K)-Akt-SREBP1c-regulated glycolysis to induce lipogenesis [47–49]. The nuclear accumulation and activation of SREBP1 are regulated by complex 1 of the mechanistic target of rapamycin (mTORC1), which is downstream of the oncogenic PI3K-Akt pathway [49, 50]. Akt stimulates SREBP1c-mediated lipogenesis via the mTOR pathway in cancer [51, 52]. We hypothesized that PC regulates SREBP1c-related fatty acid synthesis through Akt/mTOR signaling. Western blot analysis showed that after PC knockdown, the phosphorylation of Akt and mTOR was significantly reduced (shown in Fig. 5A). In addition, the PI3K inhibitor LY294002 inhibited the expression of upstream components of the Akt/mTOR signaling pathway and downregulated the protein and mRNA expression of SREBP1c and the phosphorylation of Akt and mTOR in TC cells with different PC expression levels (shown in Fig. 5B, C). The contents of intracellular TAG and neutral lipids were also reduced after treatment with LY294002 both in 8505C/PC-WT cells and 8505C/PC-sh cells (shown in Fig. 5D-F).

### *PC* facilitates 8505C cell migration and invasion by reprogramming lipogenesis in vivo and in vitro

**Fig. 6:**
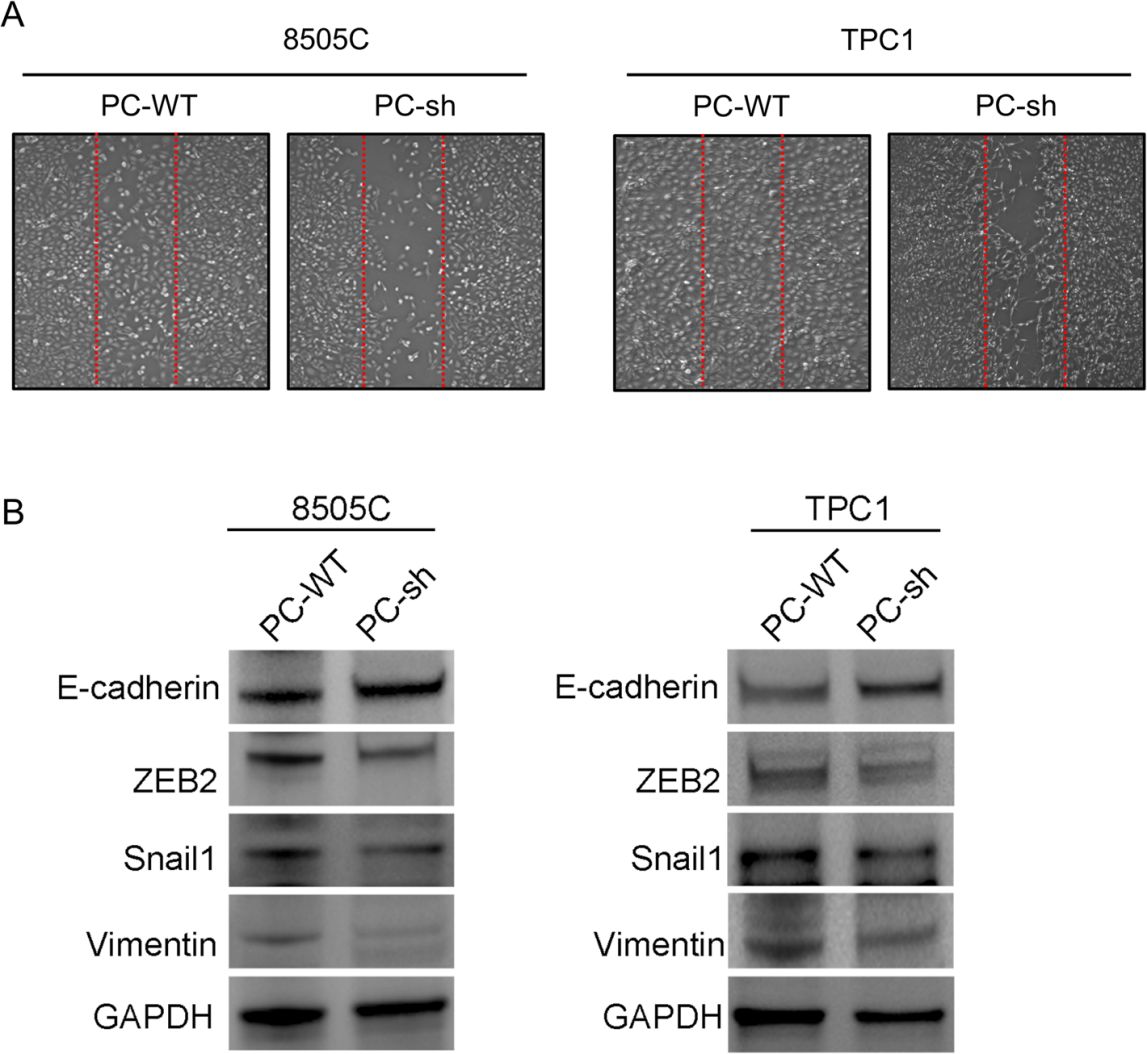
PC enhances the migration and invasion of TC cells. **A** Wound-healing assay was applied to assess the migration and invasion abilities of different 8505C and TPC1 cells (PC-WT, PC-sh) in vitro. **B** Western blot analysis of E-cadherin, Snail1, ZEB2, and Vimentin expression in TC cells compared with PC-knockdown TC cells; the full blot is provided in the supplementary file

Fatty acids have a significant association with tumor growth and metastasis. We hypothesized that PC-mediated lipogenesis is associated with thyroid tumor aggressiveness. First, the migration ability was decreased in 8505C and TPC1 cells with PC knockdown (shown in Fig. 6A). In addition, the protein levels of several transcription factors, including zeb2 and snail1, which reflect the epithelial-mesenchymal transition (EMT) of cells [53], were also significantly decreased in 8505C and TPC1 cells with PC knockdown compared to cells with normal PC expression. Furthermore, the protein expression of E-cadherin, which is a marker of cell adhesion, was upregulated in 8505C and TPC1 cells with PC knockdown compared with cells with normal PC expression, while the protein expression of Vimentin was downregulated in 8505C and TPC1 cells with PC knockdown compared with cells with normal PC expression (shown in Fig. 6B). The results suggest that PC promotes the invasion and migration of TC cells.

**Fig. 7:**
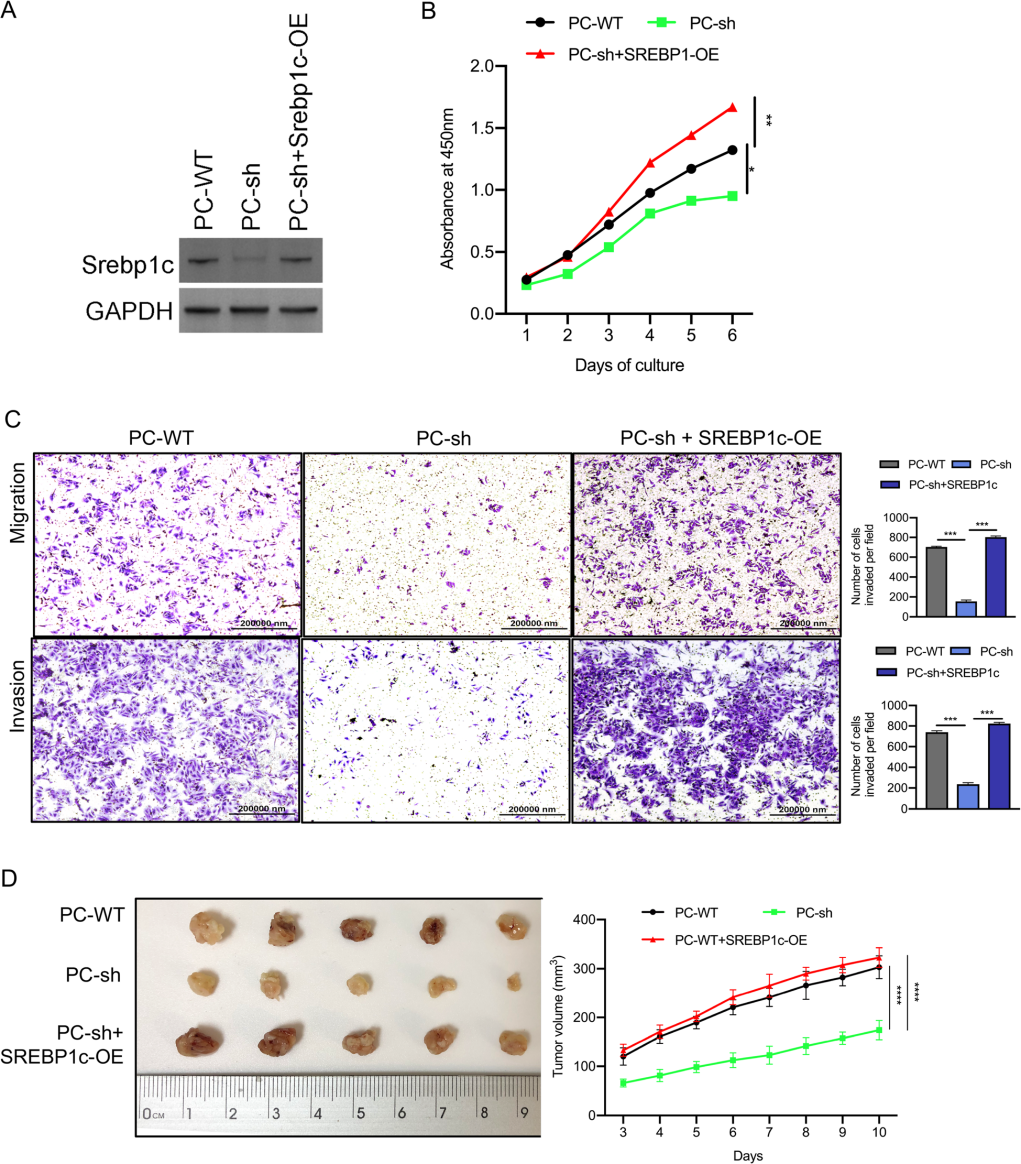
PC enhances the aggressiveness of TC cells by reprogramming fatty acid metabolism. **A** Western blot analysis of PC and SREBP1c protein expression in different 8505C cells (PC-WT, PC-sh, and PC-sh + SREBP1c-OE); the full blot is provided in the supplementary file. **B** The CCK-8 assay was used to detect the proliferation of the indicated 8505C cells. **C** Transwell invasion and metastasis assays were applied to assess the motility of different 8505C cells with a 40× objective lens. ^*^ *p* < 0.05; ^***^ *p* < 0.01. The number of invaded cells is shown in histograms on the right. **D** Tumor growth was monitored in the indicated 8505C cells, and explanted tumor images from sacrificed mice are shown. ^*^ *p* < 0.05; ^***^ *p* < 0.01. The number of invaded cells is shown in histograms on the right. Note: OE (overexpression), CCK-8 (Cell Counting Kit-8)

However, the suppressive effect on invasion and migration in 8505C/PC-sh cells was reversed after the cells were stably transfected with SREBP1c lentiviral vector (shown in Fig. 7A-C). In vitro, PC knockdown decreased tumor weight, and the effect was restored by forced expression of SREBP1c (shown in Fig. 7D). These data suggest that PC is strongly involved in the tumor aggressiveness of TC via its ability to reprogram lipogenic processes.

## Discussion

Fatty acid synthesis has been reported to be dysregulated in many cancer cells, which facilitates fatty acid biosynthesis and cell proliferation [16, 54]. In this study, we found that fatty acid synthesis also promotes TC cell progression. FASN promotes de novo fatty acid synthesis in several tumors [55–58]. SREBP1c, which is the major factor upstream of FASN, is usually activated by the AKT/mTOR signaling pathway and plays an important role in tumor cell survival and progression [41, 59–62]. However, what stimulates this pathway in TC cells remains unclear.

The PC-activated anaplerosis pathway has been explored in several cancer cells, including breast cancer, renal carcinoma, and PTC [26, 27, 29]. By bioinformatics analysis, we found that PC was overexpressed in the PTC invasive area, and assessment of clinical surgical PTC samples showed that PC was associated with lymph node metastasis. EMT is a complex and gradual process of tumor progression, and the absence of E-cadherin expression is an important marker for EMT [63, 64]. Several transcription factors, including the zinc finger proteins Snail and ZEB, which regulate cell mobility, adhesion and cytoskeletal reorganization in wound healing, inhibition of EMT [65–67]. The wound-healing assay indicated that the healing rate of the TC cell group with PC knockdown was slower than that of the TC cell control group. Western blotting results showed that the expression of E-cadherin was upregulated, while the expression of Vimentin, Snail, and ZEB2 was downregulated, indicating that PC can promote EMT of tumor cells, and inhibiting the expression of PC may prevent or slow down the invasion and migration of tumor cells. These results indicate that PC expression is correlated with TC aggressiveness. In addition, bioinformatics analysis showed that PC participates in lipid metabolic processes.

Through experimental analysis, we found that PC knockdown could significantly downregulate fatty acid synthesis as well as the expression of AKT, mTOR, SREBP1c, and FASN in TC cells. The proliferation of TC cells was also suppressed. However, the negative effect of PC knockdown was reversed by SREBP1c overexpression. Our study is the first to demonstrate that PC promotes TC progression through fatty acid synthesis. This study suggests the potential use of molecular imaging methods that could reflect the aggressive behaviors of tumors by detecting PC expression and/or lipid synthesis in living tissues and provide valuable information for clinical diagnosis and treatment.

In conclusion, our findings suggest that PC increases fatty acid synthesis and TC progression through the AKT/mTOR/SREBP1c signaling pathway.

## Supplementary Information

**Additional file 1.**

## Data Availability

The datasets used during the current study are available from the corresponding author upon reasonable request. In this study, the mRNA microarray dataset was downloaded from the Gene Expression Omnibus web portal (https://www.ncbi.nlm.nih.gov/geo/, accession number: GSE6004).

## Abbreviations

GO: Gene Ontology
DEG: Differentially expressed gene
LC/MS: Liquid chromatography/mass spectrometry
PC: Pyruvate carboxylase
TC: Thyroid cancer
TAG: Tricarboxylic acid cycle
FASN: Fatty acid synthesis
ACC1: Acetyl-CoA carboxylase
IHC: Immunohistochemistry
qRT-PCR: Quantitative reverse transcription PCR
SREBP: Sterol regulatory element-binding protein
mTOR: Mechanistic target of rapamycin
OE: Overexpression
CCK-8: Cell Counting Kit-8
