# Supplementary material for: Pyruvate carboxylase promotes thyroid cancer aggressiveness through fatty acid synthesis
Source: BMC Cancer. 2021 Jun 22;21:722. doi: 10.1186/s12885-021-08499-9 (PMC8220755; doi:10.1186/s12885-021-08499-9)
Supplement: Supplementary file 1 — Additional file 1. [file 12885_2021_8499_MOESM1_ESM.zip › Additional file1.docx]

Pyruvate carboxylase promotes thyroid cancer aggressiveness through fatty acid synthesis

Chang Liu^a^, Xiang Zhou^b^, Yu Pan^a^, Yang Liu^a^, Yifan Zhang^a*^

^a^Department of Nuclear Medicine, Ruijin Hospital, Shanghai Jiao Tong University School of Medicine, Shanghai, China

^b^Department of Nuclear Medicine, Renji Hospital, Shanghai Jiao Tong University School of Medicine, Shanghai, China

Shorting Title：Pyruvate carboxylase related fatty acid synthesis

Corresponding Author:

Yifan Zhang

Department of Nuclear Medicine, Ruijin Hospital, Shanghai Jiao Tong University School of Medicine, No. 197, Ruijin 2nd Road

Shanghai, 200025, China

E-mail: [zyf11300@rjh.com.cn](mailto:zyf11300@rjh.com.cn)


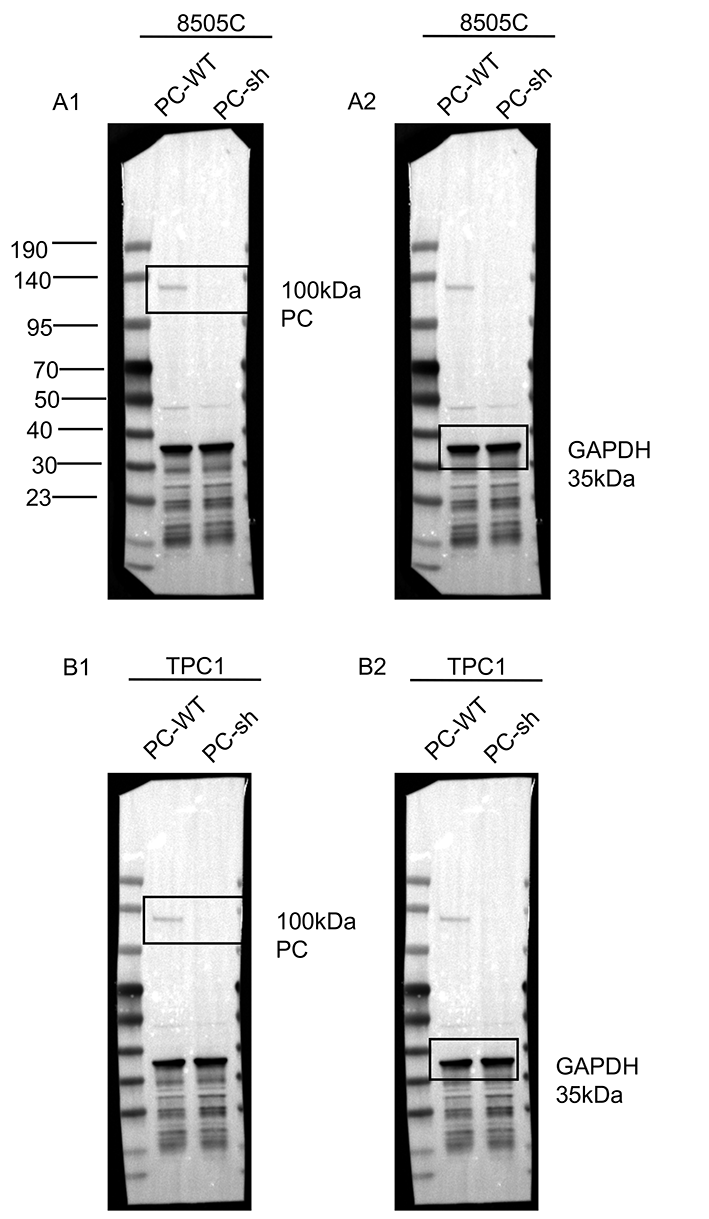


Supplementary Figure 2B. The original, full blot for PC and GAPDH expression.

A1: PC expression in 8505C cells, A2: GAPDH expression in 8505C cells, B1: PC expression in TPC1 cells, B2: GAPDH expression in TPC1 cells.


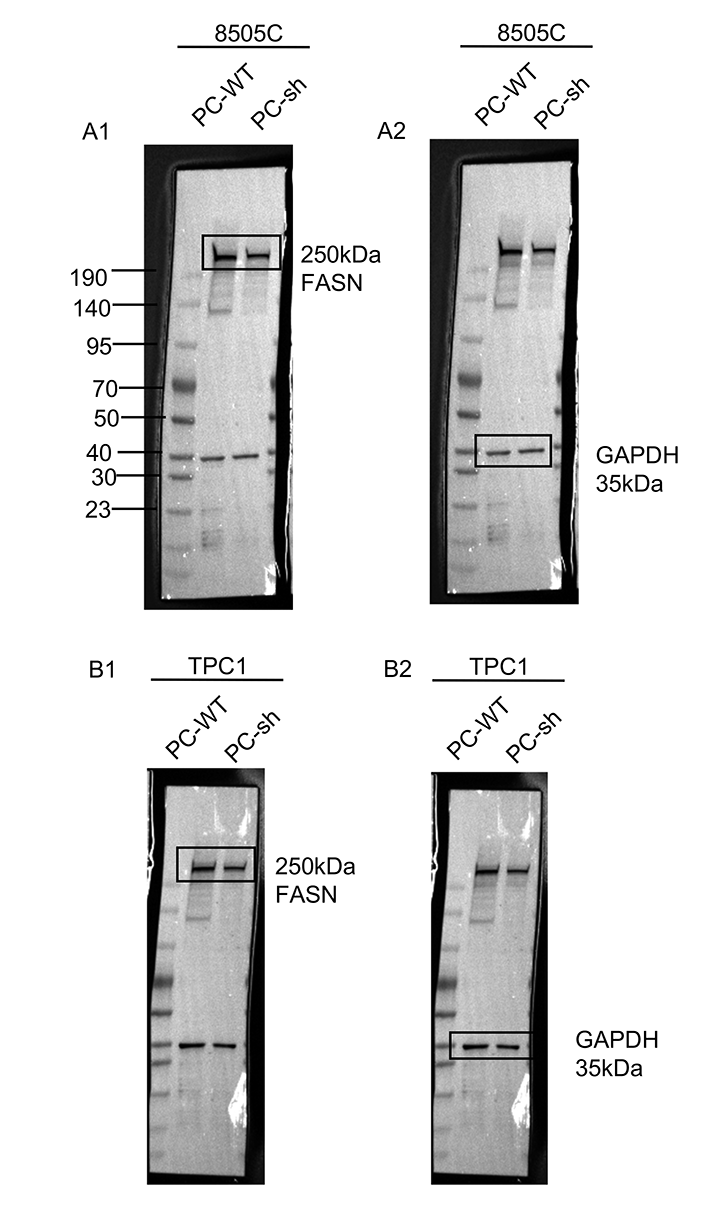


Supplementary Figure 3B. The original, full bolt for FASN, and GAPDH expression.

A1: FASN expression in 8505C cell, A2: GAPDH expression in 8505C cell, B1: FASN expression in TPC1 cell, B2: GAPDH expression n TPC1 cell.


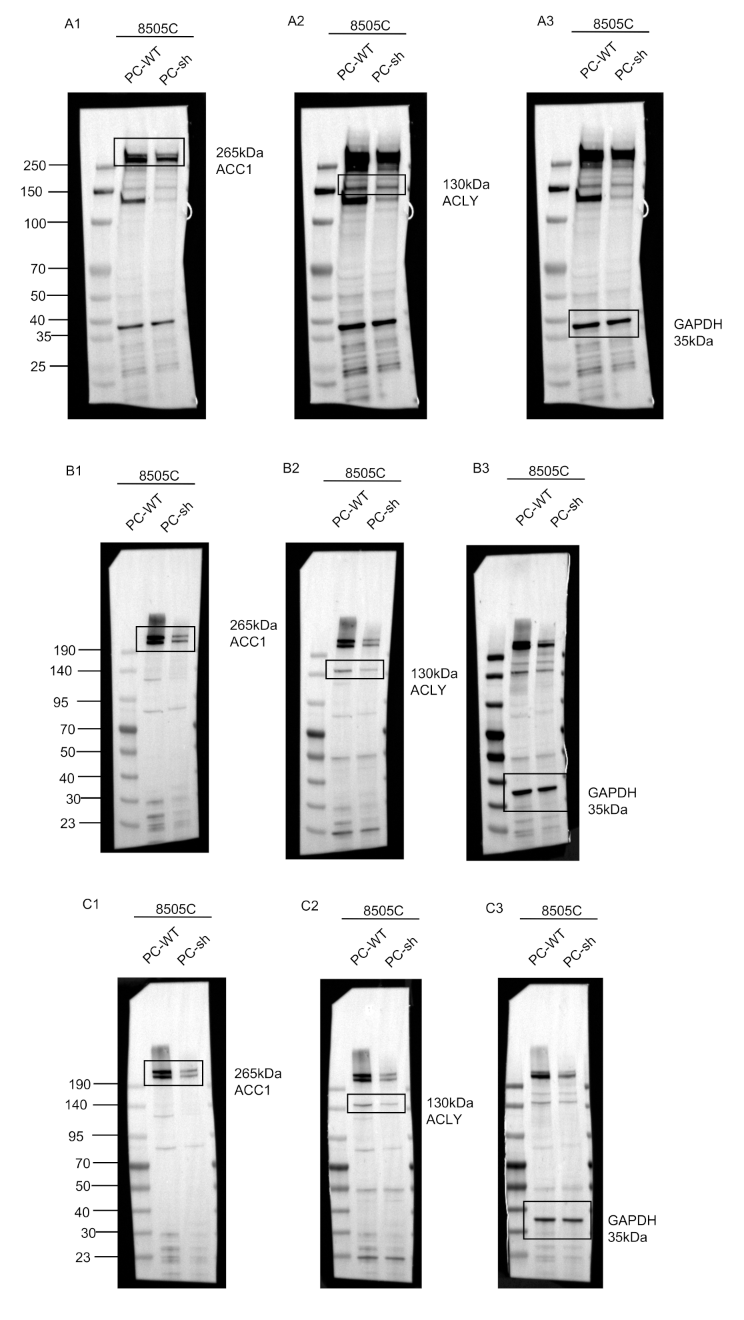


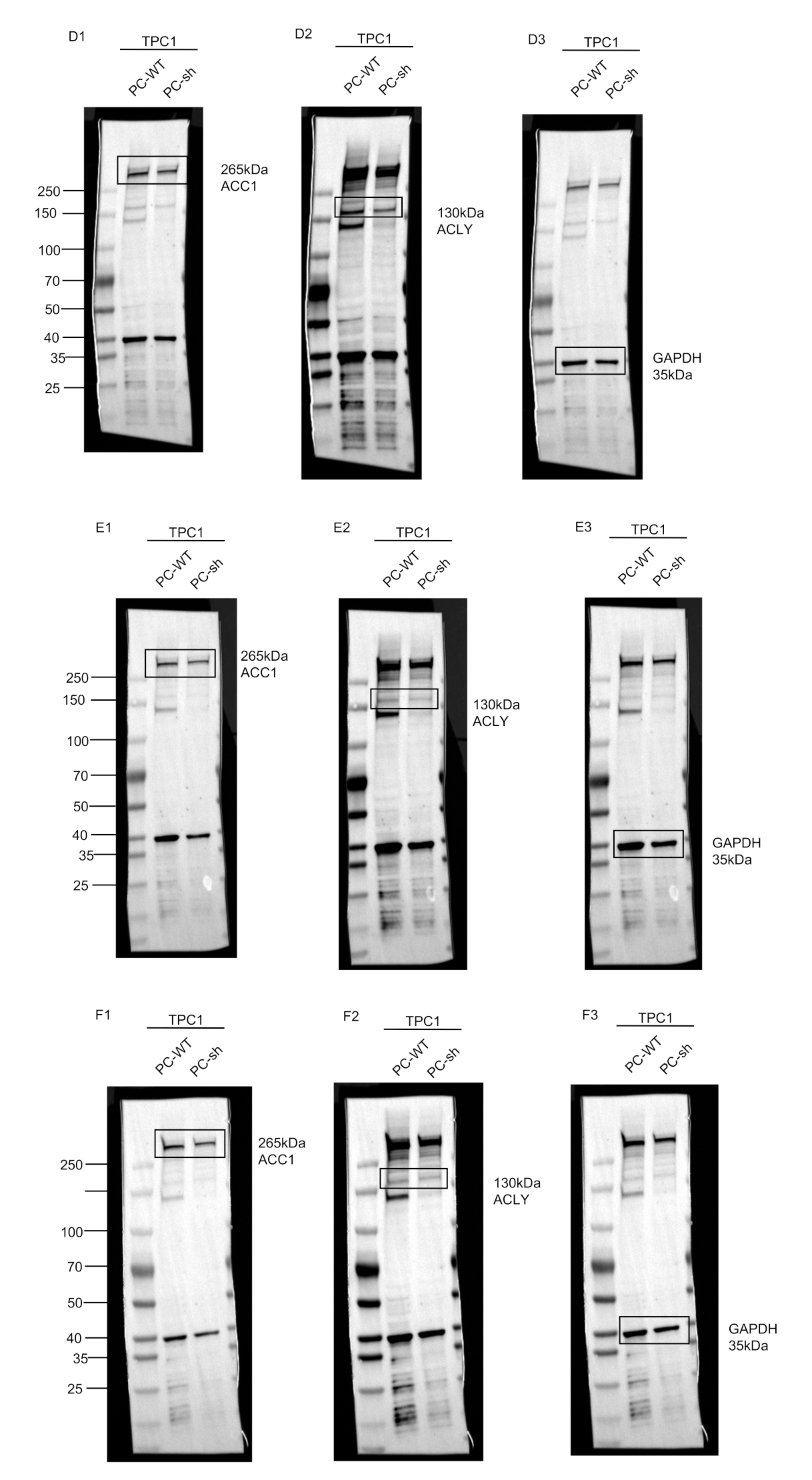


Supplementary Figure 3E. The original, full bolt for ACC1, ACLY, and GAPDH expression.

A1、B1、C1: ACC1 expression in 8505C cell, A2、B2、C2: ACLY expression in 8505C cell, A3、B3、C3: GAPDH expression in 8505C cell, D1、E1、F1: ACC1 expression in TPC1 cell, D2、E2、F2: ACLY expression in TPC1 cell, D3、E3、F3: GAPDH expression in TPC1 cell.


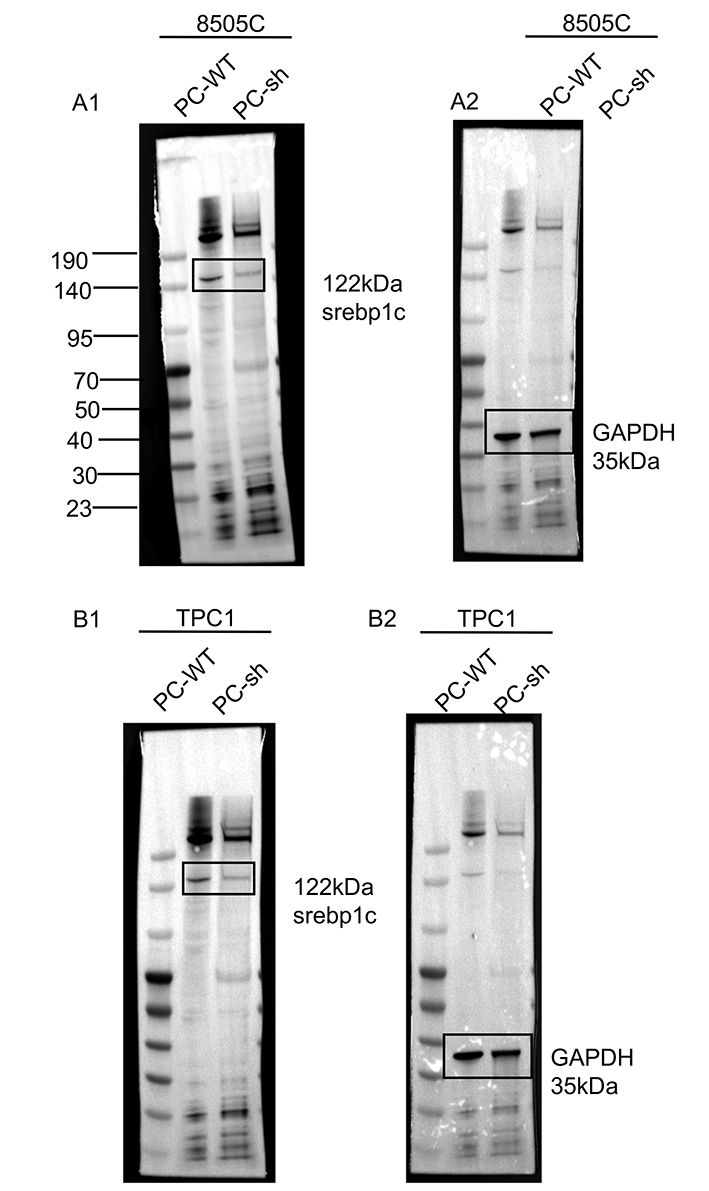


Supplementary Figure 4B. The original, full blot for SREBP1c and GAPDH expression.

A1: srebp1c expression in 8505C cells, A2: GAPDH expression in 8505C cells, B1: srebp1c expression in TPC1 cells, B2: GAPDH expression in TPC1 cells.


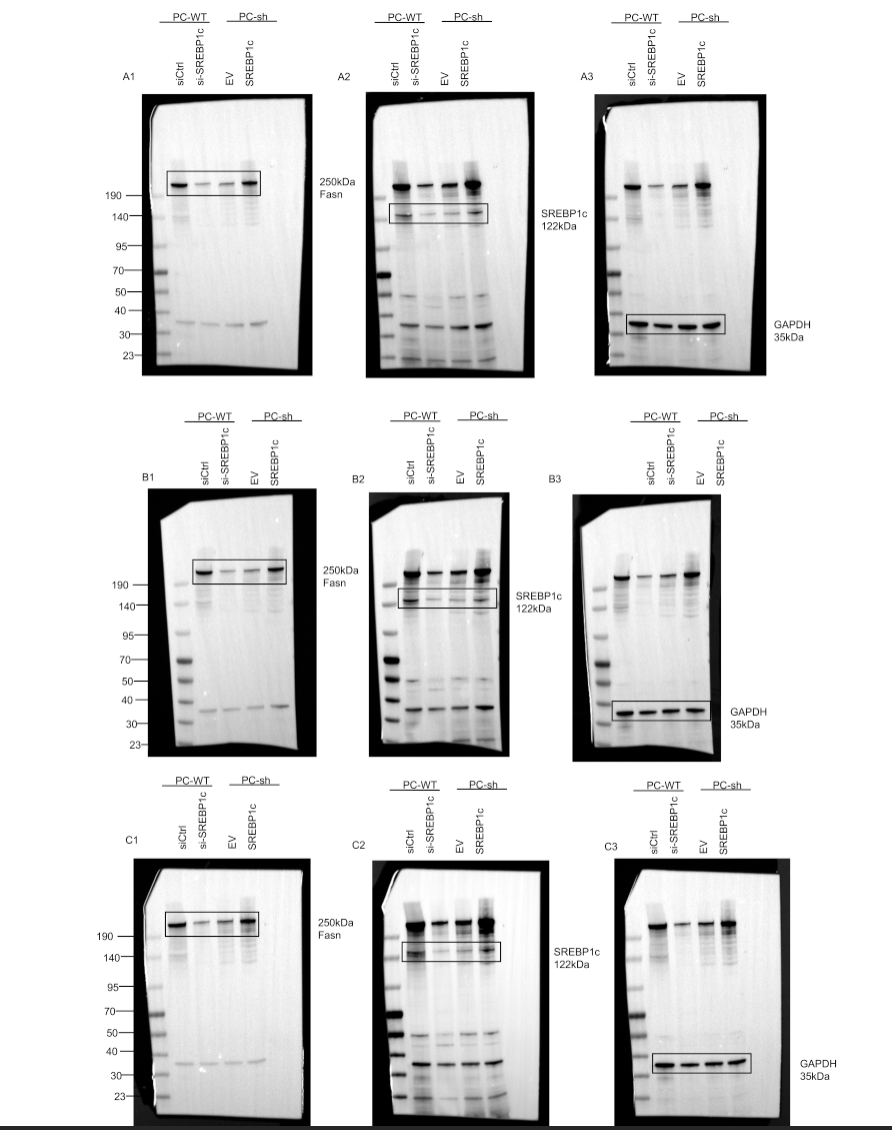


Supplementary Figure 4 E. The original, full blot for SREBP1c, FASN, and GAPDH expression.

A1、B1、C1: srebp1c expression in 8505C cells, A2、B2、C2: FASN expression in 8505C cells, A3、B3、C3: GAPDH expression in 8505C cells.


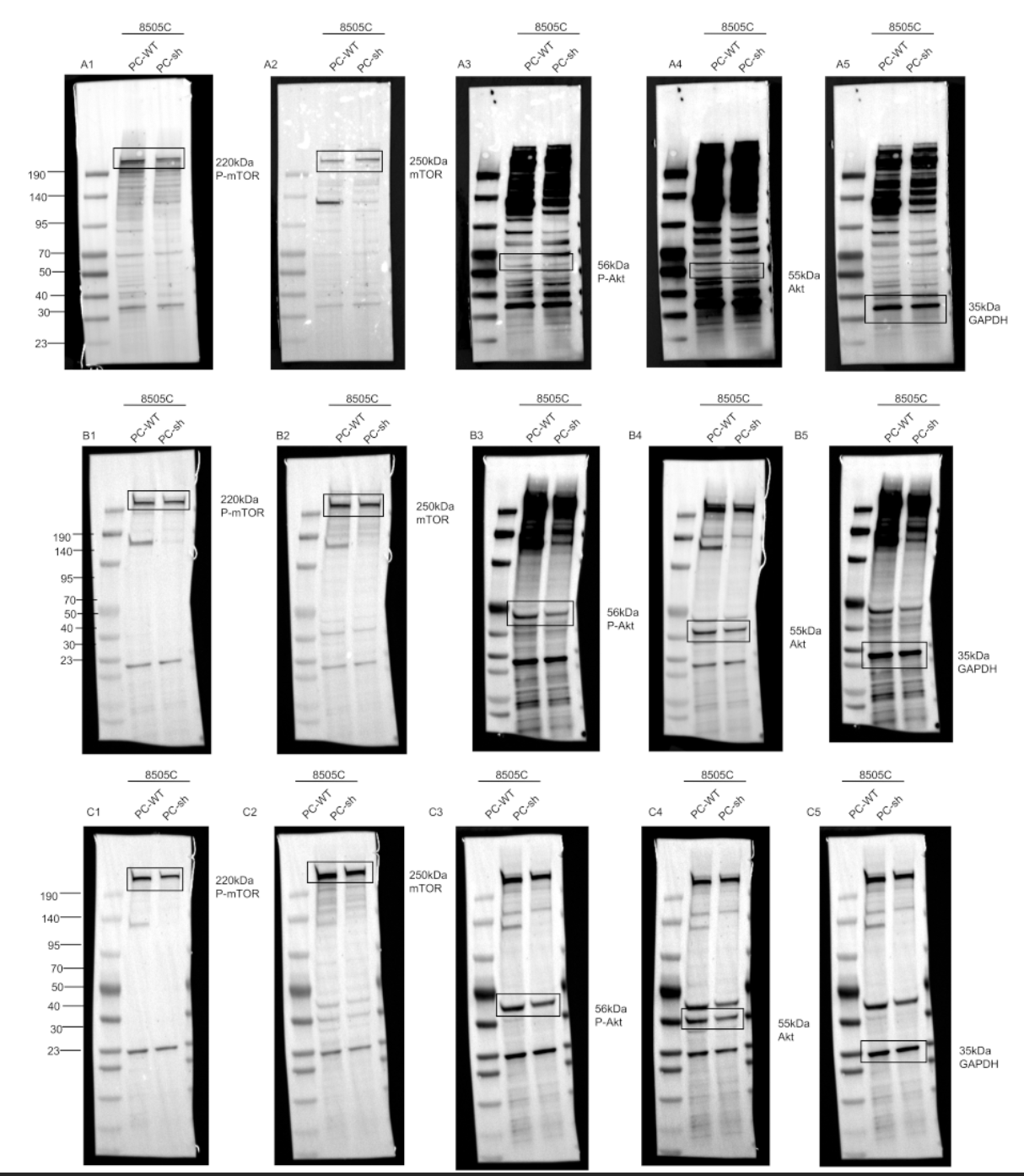


Supplementary Figure 5A. The original, full bolt for P- mTOR, PC, P-Akt (Phospho-Akt1, Ser473), Akt, mTOR, and GAPDH expression.

A1、B1、C1: P-mTOR expression in 8505C cells, A2、B2、C2: mTOR expression in 8505C cells, A3、B3、C3: P-Akt expression in 8505C cells, A4、B4、C4: AKT expression in 8505C cells, A5、B5、C5: GAPDH expression in 8505C cells.


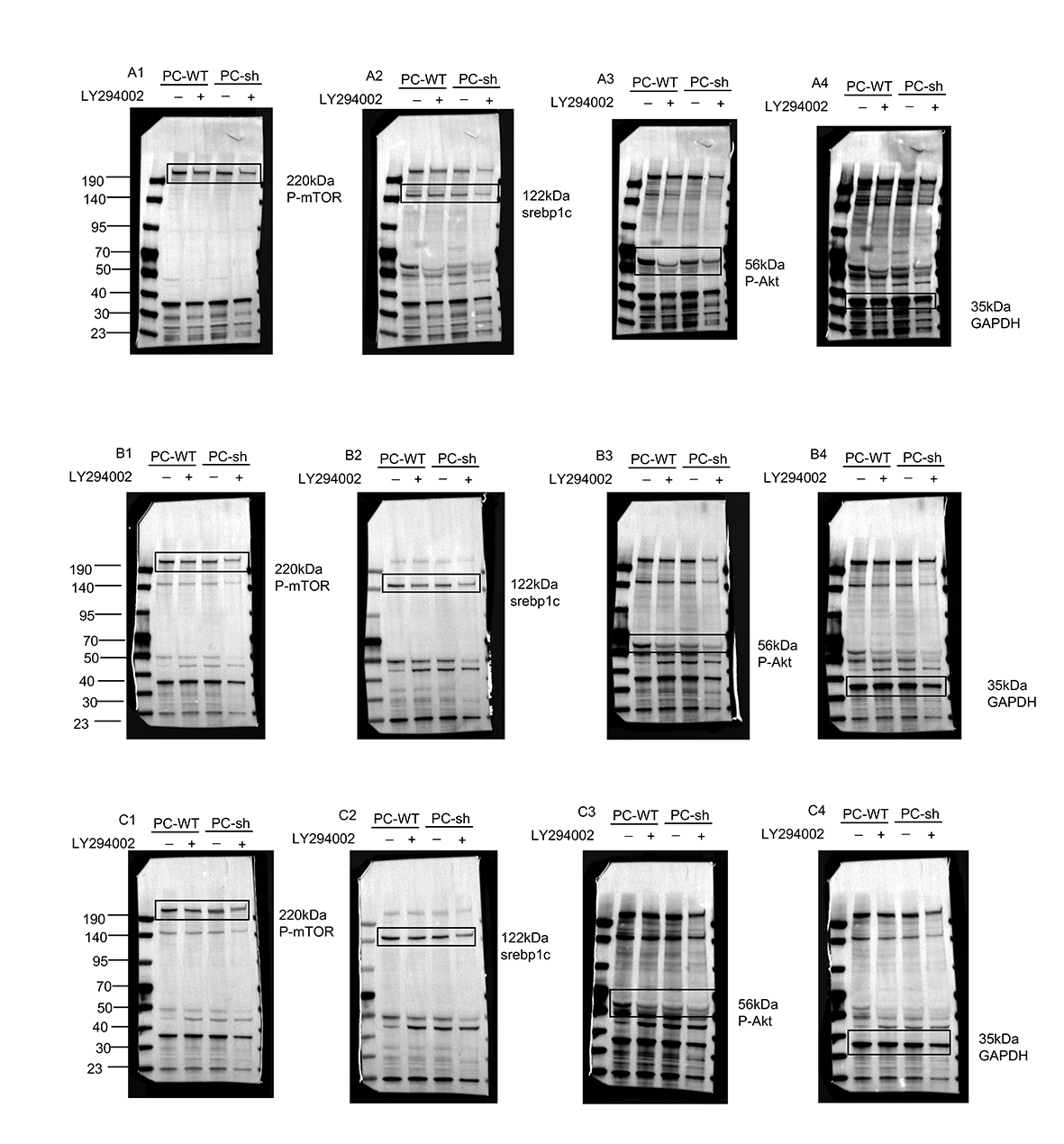


Supplementary Figure 5 C. The original, full bolt for P- mTOR, SREBP1c, P-Akt (Phospho-Akt1, Ser473), and GAPDH expression.

A1、B1、C1: P-mTOR expression in 8505C cell, A2、B2、C2: SREBP1c expression in 8505C cell, A3、B3、C3: P-Akt expression in 8505C cell, A4、B4、C4: GAPDH in 8505C cell.


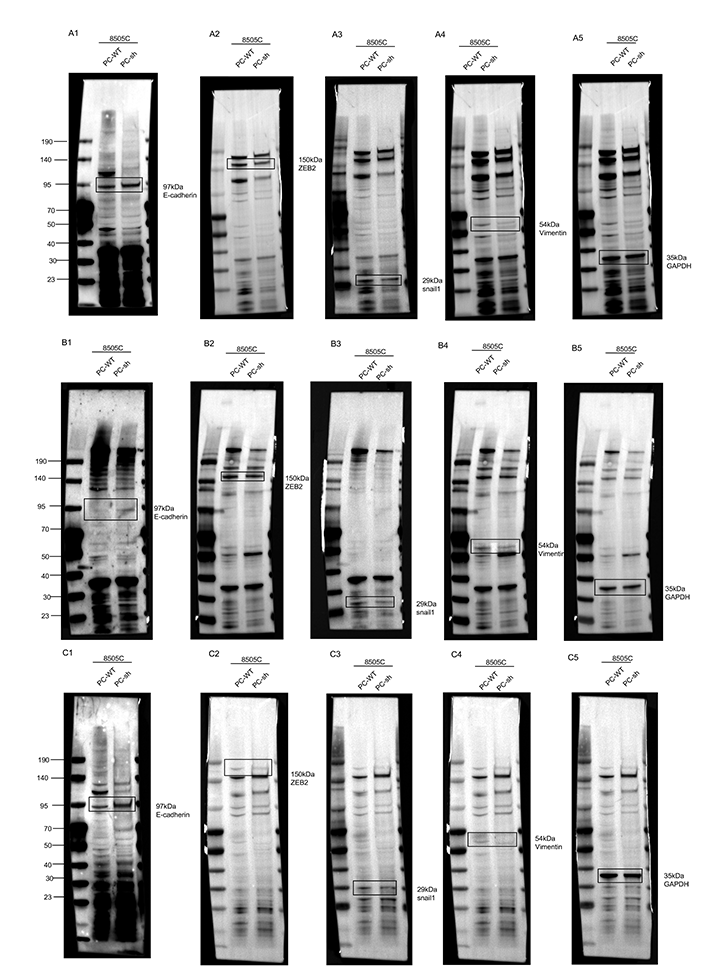


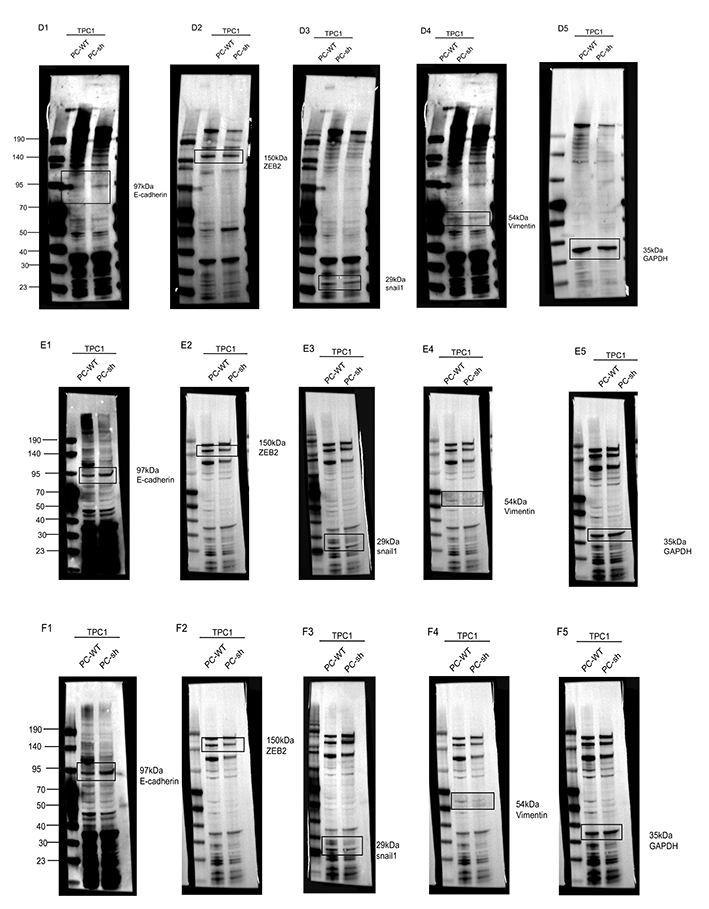


Supplementary Figure 6B. The original, full bolt for E-cadherin, ZEB2, Snail1, Vimentin, and GAPDH expression.

A1、B1、C1: E-cadherin expression in 8505C cell, A2、B2、C2: ZEB2 expression in 8505C cell, A3、B3、C3: Snail1 expression in 8505C cell, A4、B4、C4: Vimentin expression in 8505C cell, A5、B5、C5: GAPDH expression in 8505C cell, D1、E1、F1: E-cadherin expression in TPC1 cell, D2、E2、F2: ZEB2 expression in TPC1 cell, D3、E3、F3: Snail1 expression in TPC1 cell, D4、E4、F4: Vimentin expression in TPC1 cell, D5、E5、F5: GAPDH expression in TPC1 cell.


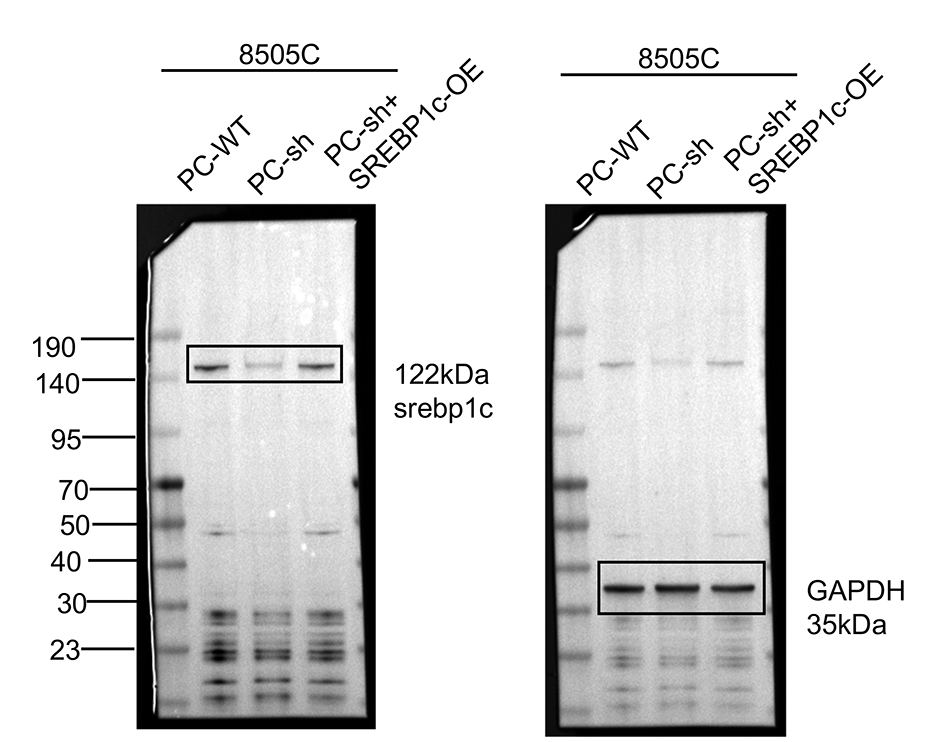


Supplementary Figure 7A. The original, full bolt for SREBP1c, and GAPDH expression.

A1: SREBP1c expression in 8505C cell, A2: GAPDH expression in 8505C cell.
