# Supplementary material for: Pyruvate carboxylase promotes thyroid cancer aggressiveness through fatty acid synthesis
Source: BMC Cancer. 2021 Jun 22;21:722. doi: 10.1186/s12885-021-08499-9 (PMC8220755; doi:10.1186/s12885-021-08499-9)
Supplement: Supplementary file 1 — Additional file 1. [file 12885_2021_8499_MOESM1_ESM.zip › Additional file2.docx]

Pyruvate carboxylase promotes thyroid cancer aggressiveness through fatty acid synthesis

Chang Liu^a^, Xiang Zhou^b^, Yu Pan^a^, Yang Liu^a^, Yifan Zhang^a*^

^a^Department of Nuclear Medicine, Ruijin Hospital, Shanghai Jiao Tong University School of Medicine, Shanghai, China

^b^Department of Nuclear Medicine, Renji Hospital, Shanghai Jiao Tong University School of Medicine, Shanghai, China

Shorting Title：Pyruvate carboxylase related fatty acid synthesis

Corresponding Author:

Yifan Zhang

Department of Nuclear Medicine, Ruijin Hospital, Shanghai Jiao Tong University School of Medicine, No. 197, Ruijin 2nd Road

Shanghai, 200025, China

E-mail: [zyf11300@rjh.com.cn](mailto:zyf11300@rjh.com.cn)


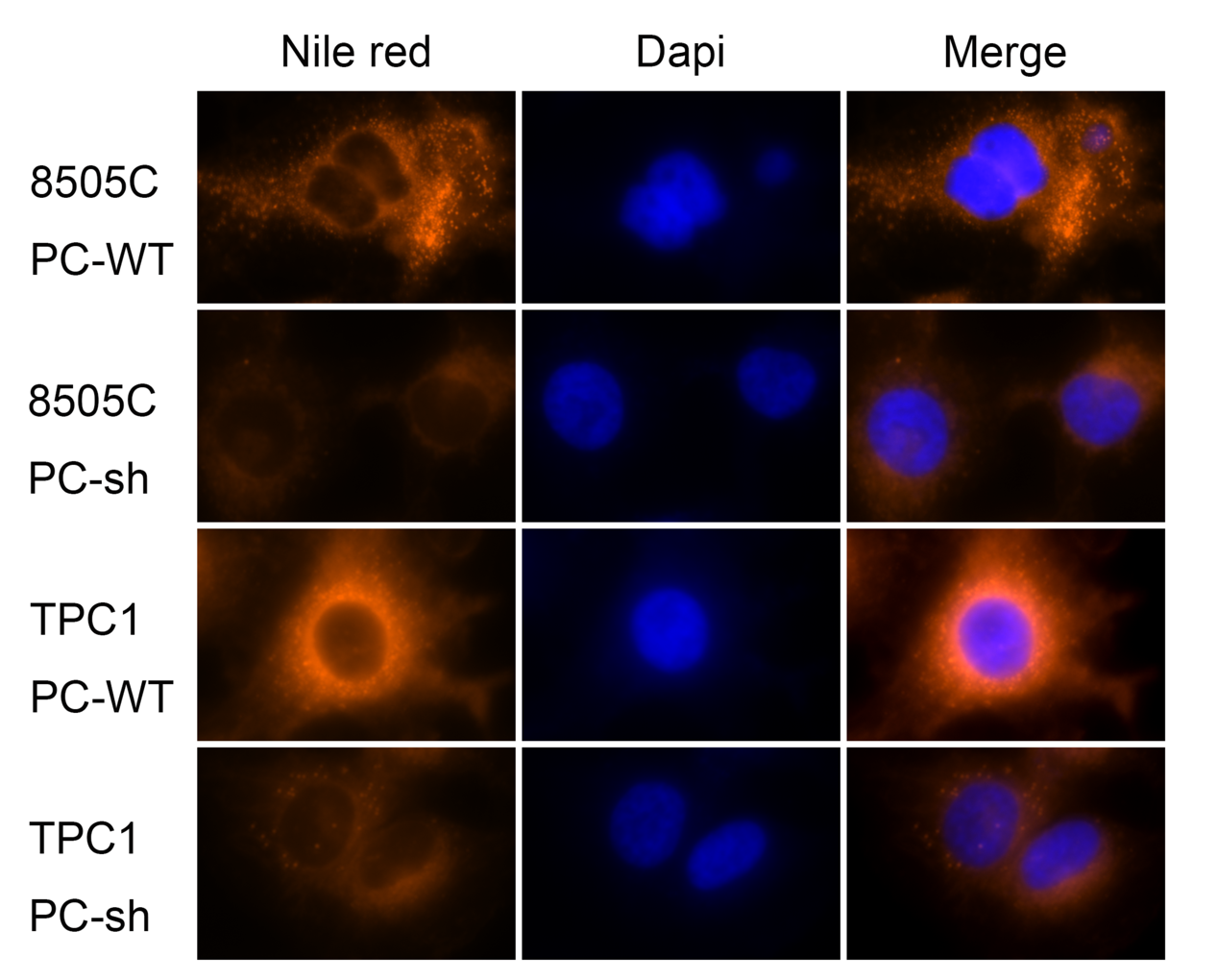


Supplementary Figure 2E: Nile red dye and DAPI staining were used to detect the content of neutral lipids in TC cells by microscopy with a 40× objective lens.


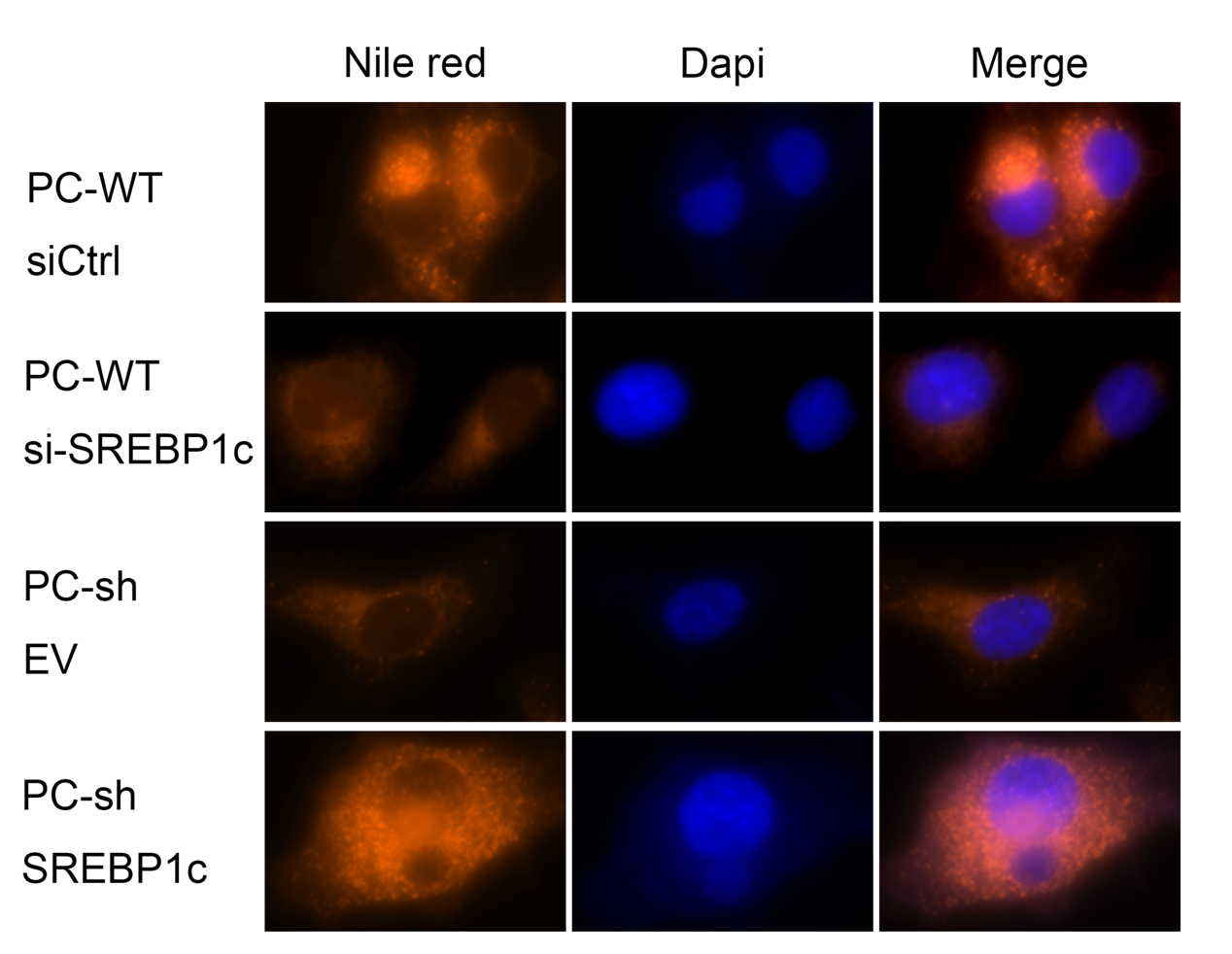


Supplementary Figure 4G: Nile red dye and DAPI staining were used to detect the content of neutral lipids in 8505C cells by microscopy with a 40× objective lens.


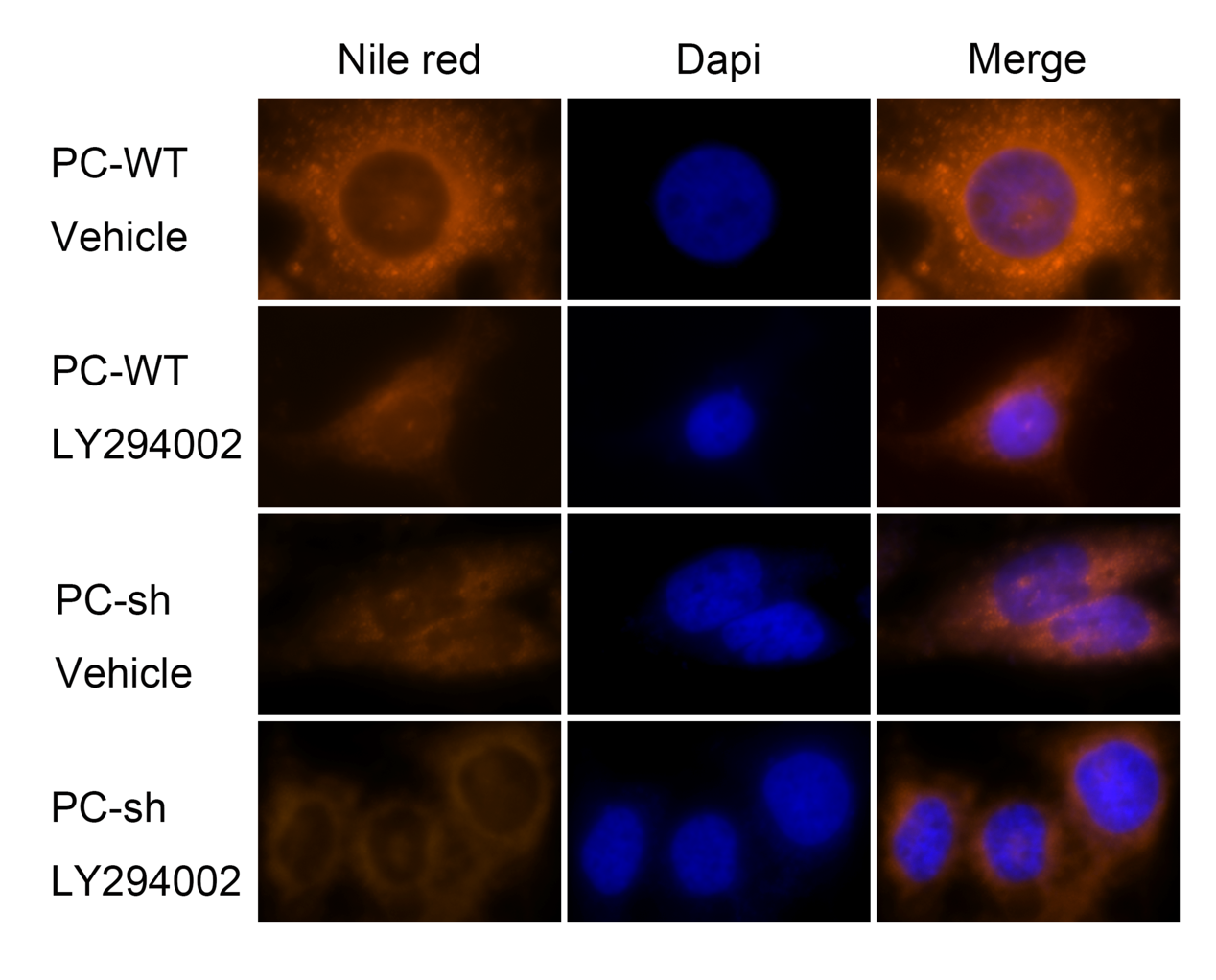


Supplementary Figure 5E: Nile red dye and DAPI staining were used to detect the content of neutral lipids in 8505C cells by microscopy with a 40× objective lens.


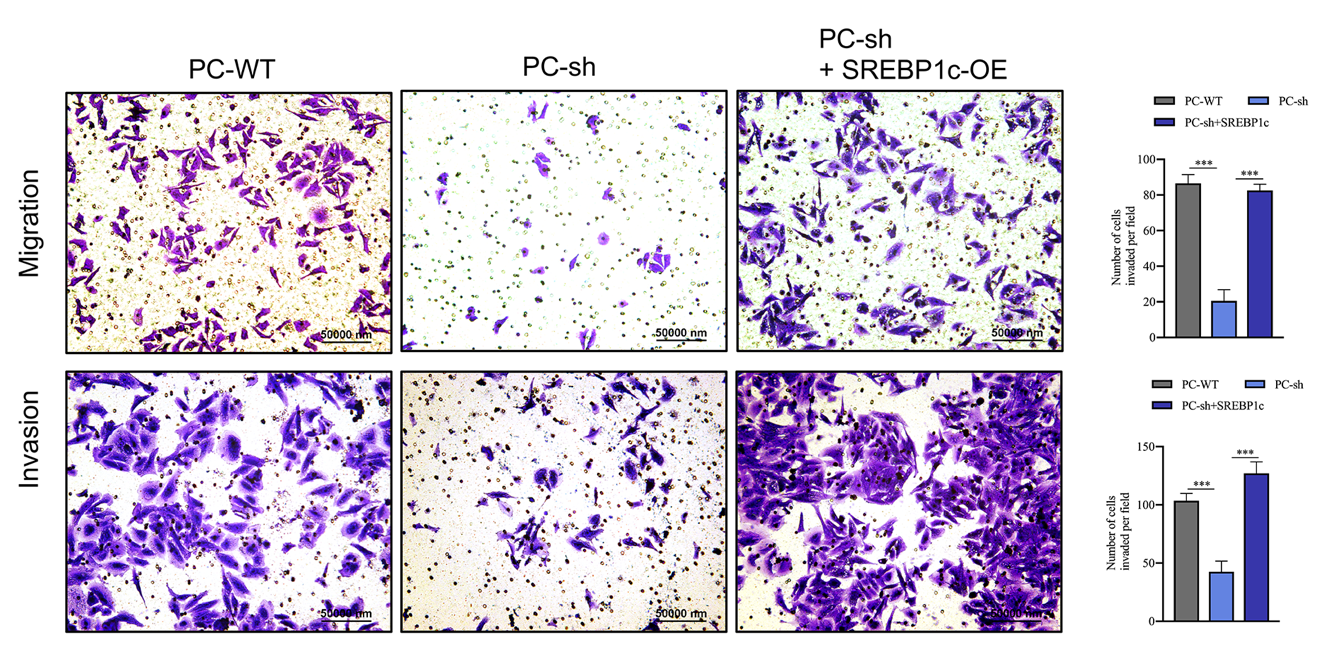


Supplementary Figure 7C: Transwell invasion and migration assays were applied to assess the motility of different 8505C cells. ^*^ *p* < 0.05; ^***^ *p* < 0.01
